# An approximate diffusion process for environmental stochasticity in infectious disease transmission modelling

**DOI:** 10.1371/journal.pcbi.1011088

**Published:** 2023-05-18

**Authors:** Sanmitra Ghosh, Paul J. Birrell, Daniela De Angelis

**Affiliations:** 1 MRC Biostatistics Unit, University of Cambridge, Cambridge, United Kingdom; 2 UK Health Security Agency, London, United Kingdom; University of Notre Dame, UNITED STATES

## Abstract

Modelling the transmission dynamics of an infectious disease is a complex task. Not only it is difficult to accurately model the inherent non-stationarity and heterogeneity of transmission, but it is nearly impossible to describe, mechanistically, changes in extrinsic environmental factors including public behaviour and seasonal fluctuations. An elegant approach to capturing environmental stochasticity is to model the force of infection as a stochastic process. However, inference in this context requires solving a computationally expensive “missing data” problem, using data-augmentation techniques. We propose to model the time-varying transmission-potential as an approximate diffusion process using a path-wise series expansion of Brownian motion. This approximation replaces the “missing data” imputation step with the inference of the expansion coefficients: a simpler and computationally cheaper task. We illustrate the merit of this approach through three examples: modelling influenza using a canonical SIR model, capturing seasonality using a SIRS model, and the modelling of COVID-19 pandemic using a multi-type SEIR model.

## Author summary

Over the course of an epidemic, the ability to transmit infection is a key parameter in the epidemic models that seek to quantify and forecast epidemic burden. This parameter will evolve considerably over time due to the emergence of new variants, changing governmental advice and access to testing and possible immunisation. Ignoring these factors can lead to models that produce overly confident estimates and biased predictions. In this paper we propose a computationally efficient method to quantifying this uncertainty in such a way that makes no assumptions about the timing and the magnitude of the impacts of these external stimuli influencing transmission. Our approach relies on the application of an approximate diffusion process to characterise changes in transmission over time. This results in a sizeable computational advantage over previous methods that use a true diffusion process, while retaining a similar quality of estimation. Our methodology would be very valuable to modellers attempting to monitor epidemics in real time.

This is a *PLOS Computational Biology* Methods paper.

## 1 Introduction

Mathematical modelling of the complex dynamics of infectious diseases remains an essential tool to inform public health policies during epidemic outbreaks. The major focus of such modelling work is describing the intrinsic transmission dynamics and the flow of individuals between compartments that segregate the population as per their disease state. However, an epidemic is also driven by a number of extrinsic factors, including population mobility, social cycles (e.g. holidays), non-pharmaceutical interventions, and climatic variations [1]. In a compartmental model, such factors are often introduced explicitly through the description of the hazard (force) of infection when information about these external drivers is available [2–4]. However, while it is impossible to fully account for all extrinsic factors influencing transmission, yet ignoring this epistemic uncertainty often known as “environmental stochasticity” leads to a structural miss-specification of the model, a “model discrepancy”. Model discrepancy can lead to miss-calibrated models that underestimate uncertainty and produce biased predictions [5]. An elegant approach to account for the un-modelled model discrepancy is to represent the force of infection as a stochastic process. For example, [6, 7] use a diffusion process for this purpose, while [8] use a discrete time stochastic process. Parameter estimation for such stochastic models is, however, challenging. Inference, particularly in a Bayesian context, requires estimation of the joint posterior distribution of both the latent path of the stochastic process and the model parameters. Estimation using a Markov chain Monte Carlo (MCMC) algorithm, involves sampling the realisation of the stochastic process, a high dimensional object, often through *data-augmentation* techniques, which incur a hefty computational cost [9]. As a result efficient calibration of a compartmental model, which embeds a stochastic process, has received significant attention in the literature [10, 11] with the goal of alleviating the computational bottleneck associated with the inference of the stochastic process.

In this paper we propose a new approach to the calibration problem through the use of a path-wise approximation of a diffusion process. Specifically, we apply a truncated Fourier expansion of a Brownian motion to obtain the approximation. Application of this series expansion turns the task of inferring a high dimensional latent diffusion sample path into the task of inferring a smaller dimensional object, the expansion coefficients, which can be carried out without data-augmentation. This method is also applicable in the context of discrete time processes that converge to a diffusion in the continuous time limit. Such processes can be approximated by first carrying out the series expansion of the limiting diffusion and then applying a suitable time discretisation.

We validate the proposed method, firstly, against a data augmentation technique carried out using a particle MCMC sampler proposed in [6], using a dataset from an influenza outbreak in a boarding school. We then further validate this approach using a model where the force of infection is depicted as a sinusoidal signal. Finally, we apply this method to fit a model of COVID-19 spread in England during the first wave.

## 2 Background: Epidemic models with a time-varying transmission-potential

We consider the canonical SIR (Susceptible-Infected-Removed) model [12] to introduce the stochastic modelling framework, although the methodology can be applied to other more complex compartmental models. In the SIR model the compartments denote the number of susceptible (*S*), infected (*I*), and recovered (*R*) people in a population subjected to an epidemic at time *t*. For a population of size *N*, the SIR model is defined by the following ODE system:

$$\begin{array}{c} \frac{dS_{t}}{dt}=-\beta S_{t}\frac{I_{t}}{N},\;\frac{dI_{t}}{dt}=\beta S_{t}\frac{I_{t}}{N}-\gamma I_{t},\;\frac{dR_{t}}{dt}=\gamma I_{t}, \end{array}$$ (1)

where $\lambda =\beta \frac{I_{t}}{N}$ is the force of infection, describing the generation of infections with a transmission-potential *β*, between susceptible individuals and the fraction, *I*_*t*_/*N*, of infectious individuals. The expected period spent in the compartment is given by *γ*^−1^. The individual compartment sizes sum to *N* = *S*_*t*_ + *I*_*t*_ + *R*_*t*_.

To include environmental stochasticity we introduce a time-varying *β*_*t*_ [13–15] to mitigate model discrepancy, leading to a reformulation of the model in Eq (1):

$$\begin{array}{c} \begin{array}{cc} dx_{t} & =a\left(x_{t},\xi_{a}\right)dt+b\left(x_{t},\xi_{b}\right)dW_{t}\\ \beta_{t} & =g(x_{t})\\ \frac{dS_{t}}{dt} & =-\beta_{t}S_{t}\frac{I_{t}}{N},\;\frac{dI_{t}}{dt}=\beta_{t}S_{t}\frac{I_{t}}{N}-\gamma I_{t},\;\frac{dR_{t}}{dt}=\gamma I_{t}, \end{array} \end{array}$$ (2)

where *x*_*t*_ follows a diffusion process described by an Itô stochastic differential equation (SDE) [16] with drift *a*(⋅), and diffusion *b*(⋅) functions parameterised by the vectors ***ξ***_*a*_ and ***ξ***_*b*_ respectively; *W*_*t*_ is a standard Brownian motion; and *g*(⋅) is a nonlinear transformation that enforces *β*_*t*_ > 0, such as exponential or inverse-logit transformation. Here we make some mild assumptions about *a*(⋅) and *b*(⋅) such as, for example, being locally Lipschitz with a linear growth bound [16] to ensure a non-explosive solution.

Inference for the stochastic model in Eq (2) within a Bayesian framework, requires inference of the latent sample path ***x*** of the diffusion *x*_*t*_, which is indirectly observed through the time evolution of the disease states: *S*_*t*_, *I*_*t*_, *R*_*t*_. This is a missing data problem that can be addressed through data-augmentation based MCMC methods [6, 10] in which a high resolution (in time) Euler-Maruyama discretisation of *x*_*t*_ is sampled along with the model parameters. Such MCMC methods incur high computational costs and have reduced efficiency in terms of mixing and speed of convergence. In what follows we will investigate a scalable approximation of *x*_*t*_ that is faster to sample.

## 3 Methods

Following [17–19], we carry out a Fourier expansion of a Brownian motion *W*_*t*_ and obtain a smooth path-wise series approximation. Using this approximation of a Brownian motion, we can in turn approximate the SDE for *x*_*t*_ with a random ODE. Inference of *x*_*t*_ can then be carried out by inferring coefficients of this ODE, without requiring data-augmentation.

### 3.1 Fourier expansion of Brownian motion

Within a time interval [0, *T*], where *T* is the length of the time horizon within which an epidemic is analysed, the Fourier expansion of a Brownian motion *W*_*t*_ is given by [18]:

$$\begin{array}{c} W_{t}=\sum_{i=1}^{\infty }(\int_{0}^{T}\phi_{i}\left(s\right)dW_{s})\int_{0}^{t}\phi_{i}\left(u\right)du. \end{array}$$ (3)

where ${\left\{\phi_{i}\right\}}_{i=1}^{\infty }$ is a complete orthonormal basis of *L*^2^[0, *T*] (see Appendix A in S1 Text for derivation). For example this can be the generalised Fourier cosine basis [20] given by

$$\begin{array}{c} \phi_{i}\left(t\right)={\left(2/T\right)}^{1/2}\text{cos}\left\{\left(2i-1\right)\pi t/2T\right\}. \end{array}$$ (4)

We will use the shorthand $Z_{i}=\int_{0}^{T}\phi_{i}\left(s\right)dW_{s}$. Since the basis functions {*ϕ*_*i*_} are deterministic and orthonormal, it follows from standard results of Itô calculus that $Z_{i}∼N\left(0,1\right)$ [18]. By truncating the infinite series in Eq (3) to *n*-terms we obtain a path-wise approximation of the Brownian motion *W*_*t*_ given by

$$\begin{array}{c} {\hat{W}}_{t}=\sum_{i=1}^{n}Z_{i}\int_{0}^{t}\phi_{i}\left(u\right)du. \end{array}$$ (5)

Following [17], if we order the basis functions by the number of times they change sign on the interval [0, *T*], then when *i* is small *Z*_*i*_ governs the low-frequency oscillations of the Brownian motion.

### 3.2 Approximating a SDE with a random ODE

Taking derivative of ${\hat{W}}_{t}$ with respect to time we obtain the following approximation to white noise, the derivative of Brownian motion, given by

$$\begin{array}{c} \frac{d{\hat{W}}_{t}}{dt}=\sum_{i=1}^{n}Z_{i}\phi_{i}\left(t\right). \end{array}$$ (6)

Now, let us replace the Itô SDE in Eq (2) with the following Stratonovich SDE [16]

$$\begin{array}{c} dx_{t}=a^{\prime }\left(x_{t},\xi_{a}\right)dt+b\left(x_{t},\xi_{b}\right)\circ dW_{t}, \end{array}$$ (7)

where (∘) denotes a Stratonovich integral [16] with respect to *W*_*t*_. The Itô SDE in Eq (2) and the Stratonovich SDE given above are equivalent [16] if

$$\begin{array}{c} a^{\prime }\left(x_{t},\xi_{a}\right)=a\left(x_{t},\xi_{a}\right)-\frac{b(x_{t},\xi_{b})}{2}\frac{\partial b(x_{t},\xi_{b})}{\partial x_{t}}b\left(x_{t},\xi_{b}\right). \end{array}$$ (8)

By substituting the term *dW*_*t*_ in Eq (7) with the approximation $d{\hat{W}}_{t}$ in Eq (6), we obtain the following (random) ODE:

$$\begin{array}{c} \frac{d{\hat{x}}_{t}}{dt}=a^{\prime }\left({\hat{x}}_{t},\xi_{a}\right)+b\left({\hat{x}}_{t},\xi_{b}\right)\sum_{i=1}^{n}Z_{i}\phi_{i}\left(t\right). \end{array}$$ (9)

The work of [21] shows that as *n* → ∞ the solution ${\hat{x}}_{t}$ of the above ODE will converge to the solution *x*_*t*_ of the Stratonovich SDE in Eq (7) which, given the choice of *a*′(⋅) in Eq (8), is an equivalent representation of the Itô SDE in Eq (2). Thus, the series approximation ${\hat{x}}_{t}$ of the solution *x*_*t*_ of an Itô SDE converges to the solution of an equivalent Stratonovich SDE.

Next, we discuss the implications of the above approximation with regards to inference.

### 3.3 Inference using the series approximation

Using the path-wise series approximation of a diffusion process *x*_*t*_, presented in the previous sections, we can re-write the canonical SIR model in Eq (2) as a system of coupled ODEs given by

$$\begin{array}{c} \begin{array}{cc} \frac{d{\hat{x}}_{t}}{dt} & =a^{\prime }\left({\hat{x}}_{t},\xi_{a}\right)+b\left({\hat{x}}_{t},\xi_{b}\right)\sum_{i=1}^{n}Z_{i}\phi_{i}\left(t\right)\\ \beta_{t} & =g({\hat{x}}_{t})\\ \frac{dS_{t}}{dt} & =-\beta_{t}S_{t}\frac{I_{t}}{N},\;\frac{dI_{t}}{dt}=\beta_{t}S_{t}\frac{I_{t}}{N}-\gamma I_{t},\;\frac{dR_{t}}{dt}=\gamma I_{t}, \end{array} \end{array}$$ (10)

where *a*′(⋅) is given by Eq (8). Note that the randomness in the above model is now encapsulated in the expansion coefficients ***Z*** = (*Z*_1_, …, *Z*_*n*_). Inference in this model is then relegated to the inference of all the parameters: ***Z***, ***ξ***_*a*_, ***ξ***_*b*_, *γ*, and the initial values: *x*_0_, *S*_0_, *I*_0_, *R*_0_. We denote the vector of the parameters governing the dynamics as ***θ*** = (***ξ***_*a*_, ***ξ***_*b*_, *γ*). We denote the state vector evolving in continuous time by ***X***_*t*_ = (*x*_*t*_, *S*_*t*_, *I*_*t*_, *R*_*t*_), and by ***X***_0_ = (*x*_0_, *S*_0_, *I*_0_, *R*_0_) the vector of initial values.

In order to explain the inferential framework based on the series approximation, in Eq (10), we assume that the available data $y_{t_{1:m}}=\left(y_{t_{1}},\ldots ,y_{t_{m}}\right)$ are the noisy observations of the state *I*_*t*_ at *m* time-points. Here we are simply considering prevalence data for the ease of exposition, however the same idea can be extended to more complex observational models where the observed data only provide partial (and often indirect) information of the states ***X***_*t*_ [8].

The inferential goal is to learn the posterior distribution of all the unknown quantities, given the data $y_{t_{1:m}}$. We place priors *p*(***θ***), *p*(***Z***), *p*(***X***_0_) on the parameters, expansion coefficients and the initial values. Note that, by construction, the ***Z*** = (*Z*_1_, …, *Z*_*n*_) have an independent standard Normal prior, see Section 3.1. We then numerically solve Eq (10) to obtain a likelihood $p(y_{t_{1:m}}|I_{t_{1:m}},\epsilon )$, based on the noise assumption, where $I_{t_{1:m}}$ is the numerical solution of the state *I*_*t*_ evaluated at the *m* time-points, and ***ϵ*** are the parameters of the chosen data distribution. The posterior distribution, up to a normalisation constant, follows from the Bayes rule:

$$\begin{array}{c} p\left(\theta ,Z,X_{0},\epsilon |y_{t_{1:m}}\right)\propto p\left(y_{t_{1:m}}|I_{t_{1:m}},\epsilon \right)p\left(\theta \right)p\left(Z\right)p\left(X_{0}\right)p\left(\epsilon \right). \end{array}$$ (11)

Samples from the posterior distribution can be obtained using MCMC. The samples of the latent approximate diffusion path $\hat{x}$ are simply the numerical solution of the ODE for ${\hat{x}}_{t}$ evaluated using samples of ***θ***, ***Z***, ***X***_0_ from the posterior distribution.

Note that if we had described *x*_*t*_ using a SDE, as in Eq (2), then we had to infer the sample path of the diffusion along with the parameters. For this we had to discretise the path of *x*_*t*_ using, say, the Euler-Maruyama scheme [22]. Specifically, we had to introduce *l* points between each pair of successive observation times *t*_*i*_ and *t*_*i*+1_ (i = 0,1,…,m-1) to allow sufficient accuracy of the Euler-Maruyama scheme. Thus, for a step of *δ* = 1/(*l* + 1) we would have ended-up with a discrete skeleton $x=(x_{0},x_{\delta },x_{2\delta },\ldots ,x_{t_{m}})$, representing the sample path. This sample path ***x*** along with the parameters ***θ*** can then be estimated using a data-augmentation based MCMC algorithm, where the path ***x*** ∼ *p*(***x***|***y***, ***θ***) is preferably drawn using a particle filter as proposed in [6]. If we now contrast this inference scheme, where *x*_*t*_ is described as a SDE, to the case where *x*_*t*_ is described by an ODE (after applying the series expansion), it can be seen that as long as *n* = dim(***Z***) is chosen to be substantially smaller than dim(***x***), inference using the series expansion will be a simpler problem as we have to deal with the estimation of fewer random variables. Below we show, empirically, that choosing a value of *n* substantially smaller than dim(***x***) still renders an estimate of the posterior distribution that is a reliable approximation to the true posterior.

## 4 Evaluation

To evaluate the proposed approximation method we fit the model in Eq (10) to the data of an outbreak of influenza at a boarding school [23], on the number of infections for a period of *T* = 14 days among a population of size *N* = 763. This dataset is publicly available in the R package outbreaks [24]. This dataset was previously used in [25, 26] to fit a SIR model with a time-varying transmission-potential *β*_*t*_ under the assumption that *β*_*t*_ evolves as a stationary stochastic process, whose dynamics can be modelled using an Ornstein–Uhlenbeck (OU) SDE (since the solution of this SDE evolves as a stationary process). If we describe the dynamics of *x*_*t*_, in Eq (2), through the OU SDE

$$\begin{array}{c} dx_{t}=\left(\xi_{1}-\xi_{2}x_{t}\right)dt+\xi_{3}dW_{t}, \end{array}$$ (12)

where (*ξ*_1_ − *ξ*_2_*x*_*t*_) is the linear drift function and *ξ*_3_ is a standard deviation parameter, commonly referred to as the *volatility* parameter, representing a constant diffusion function, then the stochastic model in Eq (2) becomes similar to the model in [25]. We can re-write the model in Eq (2) as follows:

$$\begin{array}{c} \begin{array}{cc} dx_{t} & =\left(\xi_{1}-\xi_{2}x_{t}\right)dt+\xi_{3}dW_{t}\\ \beta_{t} & =\text{exp}(x_{t})\\ \frac{dS_{t}}{dt} & =-\beta_{t}S_{t}\frac{I_{t}}{N},\;\frac{dI_{t}}{dt}=\beta_{t}S_{t}\frac{I_{t}}{N}-\gamma I_{t},\;\frac{dR_{t}}{dt}=\gamma I_{t}, \end{array} \end{array}$$ (13)

where, following [25] and [26], the logarithm of the transmission-potential evolves as a stationary stochastic process.

Here we specifically want to compare the outcome of inference using the true OU diffusion used above (**SDE**) with its series approximation (**SA**), leading to a model such as in Eq (10), where the dynamics of ${\hat{x}}_{t}$ is given by the following ODE:

$$\begin{array}{c} \frac{d{\hat{x}}_{t}}{dt}=\left(\xi_{1}-\xi_{2}x_{t}\right)+\xi_{3}\sum_{i=1}^{n}Z_{i}\phi_{i}\left(t\right), \end{array}$$ (14)

where we have chosen the generalised Fourier basis in Eq (4) as the function *ϕ*_*i*_(*t*).

For the **SDE** model the latent sample path ***x***, the OU SDE parameters ***ξ***, initial value *x*_0_ and the parameter *γ* were also estimated together with the initial susceptibility, *s*_0_ = *S*(*t* = 0)/*N*, assuming the initial recovered fraction *r*_0_ = 0 and thus *i*_0_ = 1 − *s*_0_. As this is count data we have specified a Poisson likelihood:

$$\begin{array}{c} y_{t_{i}}|\theta ,x,X_{0}∼\text{Poisson}\left(I_{t_{i}}\right),\;i=1,\ldots ,m, \end{array}$$ (15)

where in this case ***X***_0_ = (*x*_0_, *s*_0_) and ***θ*** = (***ξ***, *γ*). For the **SA** model we used the inferential framework introduced in the previous section and used the Poisson likelihood as above:

$$\begin{array}{c} y_{t_{i}}|\theta ,Z,X_{0}∼\text{Poisson}\left(I_{t_{i}}\right). \end{array}$$ (16)

We chose a weakly-informative prior for the parameters governing the dynamics *ξ*_1_, …, *ξ*_3_, *γ* ∼ Γ(2, 2). For *s*_0_ a Beta(2, 1), since we expect the true value to be near or greater than 2/3, and for the initial value of the diffusion we used a prior $x_{0}∼N(\xi_{1}/\xi_{2},{\left(\frac{\xi_{3}^{2}}{2\xi_{2}}\right)}^{2})$, which is the stationary distribution of the OU diffusion.

For the **SDE** model, data-augmentation using a particle filter was employed to sample the ‘true’ diffusion’s path, following [6], and produce an unbiased estimate of the likelihood. Parameters *γ*, ***ξ***, ***X***_0_ were estimated using the Metropolis-Hastings (MH) algorithm, with an adaptive random-walk proposal based on algorithm 4 of [27]. See Appendix B in S1 Text. for further details on this proposal mechanism. The likelihood estimate produced by the particle filter was used in the acceptance step of the MH algorithm. This particle-marginal Metropolis-Hastings (PMMH) MCMC scheme for jointly updating the latent diffusion path along with the parameters has been shown to have superior performance [6] when compared to other data-augmentation approaches. For the PMMH, we used a Bootstrap particle filter [28], where the particles are propagated using Euler-Maruyama discretisation, and set the number of particles to 1000. Following [25], we carried out the Euler-Maruyama iterations with a stepsize *δt* = 0.1, leading to *l* = 9 time-points between two observations.

For the **SA** model we used the Metropolis-Hastings algorithm with the same adaptive random-walk proposal (RWMH) used with the PMMH scheme and the Euler method to numerically solve the ODE adopting the same step-size that is used with the Euler-Maruyama scheme for the SDE.

Note that inference for the **SDE** model using PMMH will be substantially more computationally heavy compared to the inference for the ODE based **SA** model, irrespective of the value of *n*. This is due to the particle filter requiring multiple evaluation of the Euler-Maruyama scheme at each MCMC iteration. Even when parallelised, the particle filter will be bottlenecked by a weight-updating step (see [28] for details) requiring message-passing across processes. The Euler scheme for solving the ODE in Eq (14), in comparison, is evaluated once every iteration of a Metropolis-Hastings algorithm targeting the posterior distribution in Eq (11).

**Fig 1:**
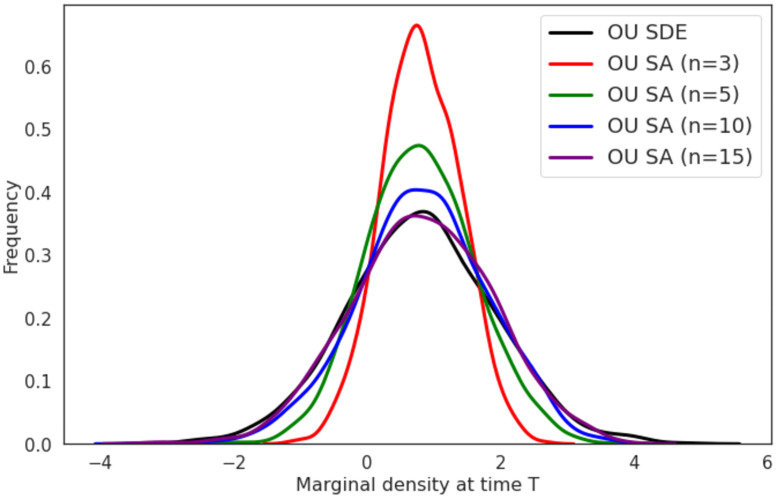
Comparison between the marginal density of the OU SDE at time *T* = 14, with that obtained through the series approximation upon varying the number of basis *n* = 3, 5, 10, 15.

A crucial parameter for the proposed method is the number of basis functions *n*. If a value of *n* produces a close match between the marginal densities of the true and approximate diffusion at the end of the analysis period *T* then the approximation will be valid throughout the course of the epidemic. In this case *T* = 14. In Fig 1 we compare the time *T* marginal densities $p\left({\hat{x}}_{t}\right){|}_{t=T}$ obtained by solving the ODE in Eq (14) associated with the **SA**, and *p*(*x*_*t*_)|_*t*=*T*_ obtained from the original OU diffusion, both based on some trial parameters sampled from the prior. The value *n* = 15 produces a close match between the marginal densities. We defer further discussion of the effect of *n* on estimation to section 4.2.

### 4.1 Results: Comparison between true and approximate diffusion

We fitted the two models, **SDE** and **SA** respectively, using the associated algorithms as described above to the influenza dataset. We ran two chains of both PMMH, for the **SDE** model, and RWMH, for the **SA** one, for 10^6^ iterations where the first 5 × 10^5^ iterations were discarded as burn-in and the remaining samples thinned to obtain 1000 samples from the posterior distribution. The running times were 15907 and 2397 seconds for the PMMH and RWMH with *n* = 15, respectively. We implemented a vectorised particle filter and the Euler solver for the ODE using Jax [29]. The adaptive MCMC algorithm was implemented using Python.

**Fig 2:**
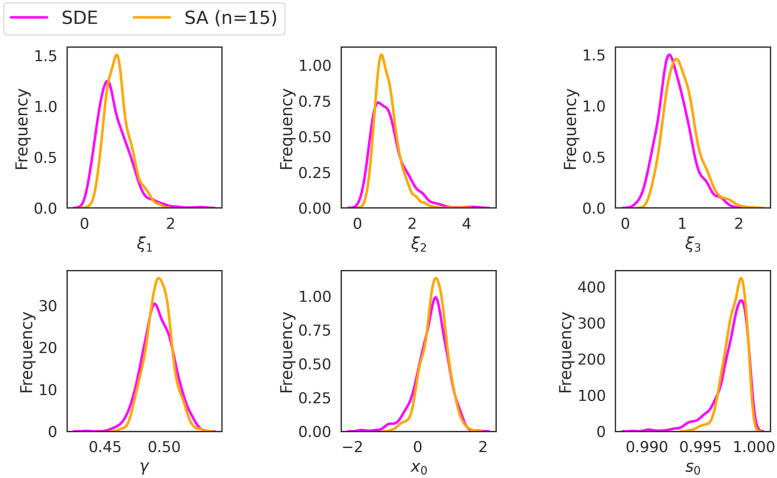
Comparison of the posterior marginal densities of the parameters obtained using the SDE and the SA (with *n* = 15 basis function). These densities are summarised using a kernel density estimate.

**Fig 3:**
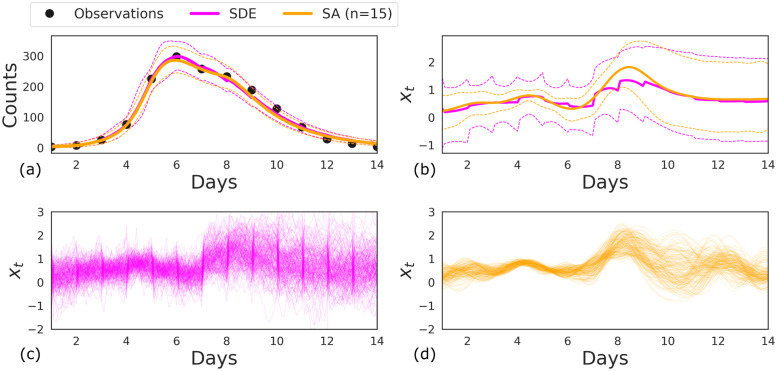
**Influenza dataset**: Goodness-of-fit (a); posterior distribution of the latent diffusion paths corresponding to the **SDE** and **SA** counterparts (b), with densities summarised by the mean (solid lines) and 95% credible intervals (broken lines); and samples from the posterior distribution of the latent diffusion paths, **SDE** (c) and **SA** (d).

We notice a good agreement between the parameter estimates obtained using the **SDE** and **SA** counterparts (see Fig 2). Furthermore, in Fig 3 we compare the goodness-of-fit and display the posterior distribution of the latent diffusion paths $p(x|y_{t_{1:m}})$ and $p(\hat{x}|y_{t_{1:m}})$, corresponding to the **SDE** and **SA**. Additionally, for aid of visualisation, we have also plotted draws from the (posterior) sample paths for both models in Fig 3(c) and 3(d).

We observe a good agreement between the epidemic curves obtained using the **SDE** and the **SA**, but for the posterior distribution of the latent diffusion paths the credible intervals are narrower for the **SA**. The **SA**, due to the truncation of the infinite series expansion, produces smoother paths, slightly underestimating the variability in the evolution of the latent diffusion path. On a closer introspection of the posterior means (Fig 3(b)), it is noticeable that the latent diffusion paths drop and increase again in the period between the 4-th and 9-th day, around the peak, indicating an increase in transmissibility. These changes are reflected in the estimates of both **SDE** and **SA**. After the 9-th day, the variability in the latent paths increase for both **SDE** and **SA** and the posterior means match closely. This is expected since after the peak, when the epidemic is receding, a large change in *β*_*t*_ will have negligible effect on the case counts. Note that there is also a considerable amount of uncertainty about the **SA**’s estimate of the maximum value of the transmission-potential around the 9-th day (see the sample paths in Fig 3(d)). That is the **SA** is uncertain whether the maximum value of the transmission-potential occurs on the 8-th or the 9-th day. This increased uncertainty, in turn, results in the credible intervals of SA matching the credible intervals of SDE after day 9.

These results were confirmed in a simulation study where the simulated datasets mimicked this influenza dataset (see Appendix C in S1 Text).

Additionally, we have fitted the model while using a negative binomial likelihood to allow overdispersion. Details are given in Appendix I in S1 Text.

### 4.2 The effect of *n* on the convergence of the posterior

In Fig 1 we noticed that the marginal distribution of the latent diffusion path and its series approximation starts agreeing beyond *n* ≥ 10 terms. It is worth investigating whether such a threshold exist for the posterior distributions obtained using the **SDE** and the **SA**. We did this by further comparing the joint posterior distribution *p*(***θ***, ***X***_0_|***y***), from **SDE** and **SA** while varying *n*. Note that ***θ*** and ***X***_0_ are quantities which were estimated using both the **SDE** and **SA** counterparts, and thus the joint posterior of these were chosen for comparison. For this comparison we estimated the posterior distribution by fitting the **SA** repeatedly with number of basis set to *n* = 3, 5, 10, 15, 20, 25, 30. To compare the posterior distributions, we used the maximum mean discrepancy (MMD) metric [30], a divergence metric that can be calculated using samples from the distributions. See Appendix H in S1 Text for further details on this metric.

**Fig 4:**
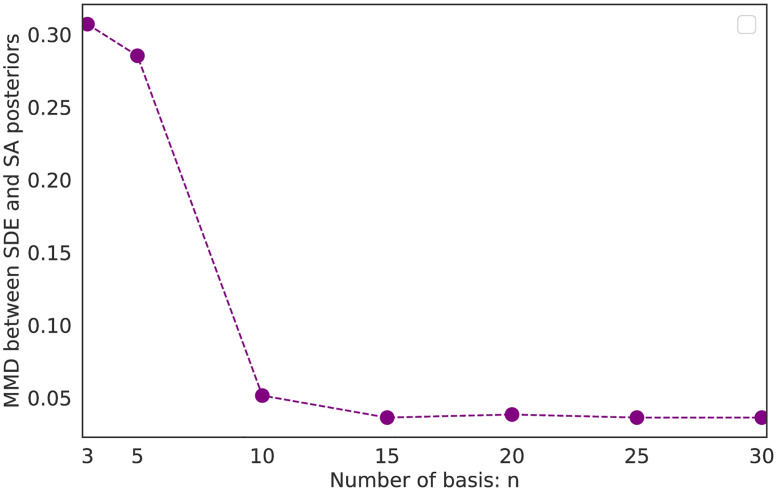
MMD between the joint posterior distributions of the parameters *θ* and initial values *X*_0_ from SDE and SA (for different *n*).

In Fig 4 we plot the MMD between the posteriors from **SDE** and **SA** for increasing *n*. For *n* ≥ 10 we found good agreement between the two posteriors, consistent with the results from comparing the marginal densities (Fig 1). This reinforces our approach of choosing the number of basis by comparing marginals of the latent process, while using the **SA**. We summarise the runtimes of MCMC with the RWMH proposal for each choice of *n* in Table 1, noting that the increase in the runtimes as we varied *n* was negligible, especially when compared to the PMMH with **SDE**.

**Table 1:** Runtimes (rounded to nearest integer), in seconds, of MCMC for SA, as a function of the number of basis *n*, in comparison with the runtime of SDE. These were run on a 3.6 GHz machine with 16 GB memory.

Runtimes in seconds								
SA with	*n* = 3	*n* = 5	*n* = 10	*n* = 15	*n* = 20	*n* = 25	*n* = 30	SDE
	2280	2282	2337	2397	2470	2538	2637	15907

## 5 Approximate diffusion for capturing a complex signal

In the previous section, when fitting the SIR model to an influenza dataset, we considered a stationary stochastic process representing the evolution of the transmission-potential. To further demonstrate the usefulness of the proposed series approximation technique we investigate here the capability of the approximate diffusion process model to recover a complex signal representing the time evolution of the transmission-potential. To this end, we follow closely the experiment furnished in [7], where observations were simulated from a SIRS model whose time-varying transmission potential was given by a deterministic sinusoidal signal. More specifically, we consider the SIRS model given by [7]

$$\begin{array}{c} \begin{array}{cc} \beta_{t} & =\beta_{0}\left(1+\beta_{1}\text{sin}\left(2\pi t/365+2\pi \beta_{2}\right)\right)\\ \frac{dS_{t}}{dt} & =\mu \left(N-S_{t}\right)-\beta_{t}S_{t}\frac{I_{t}}{N}+\alpha R_{t}\\ \frac{dI_{t}}{dt} & =\beta_{t}S_{t}\frac{I_{t}}{N}-\left(\gamma +\mu \right)I_{t}\\ \frac{dR_{t}}{dt} & =\gamma I_{t}-\left(\alpha +\mu \right)R_{t}\\ \frac{dC_{t}}{dt} & =\beta_{t}S_{t}\frac{I_{t}}{N}, \end{array} \end{array}$$ (17)

where *C*_*t*_ gives the number of new cases, 1/*α* is the average duration of immunity, *γ* is the recovery rate and *μ* is the recruitment or mortality rate. Observation $y_{t_{1:m}}$, from this model, were simulated on a discrete time-grid *t*_1:*m*_ = (*t*_1_, *t*_2_…, *t*_*m*_), with observation interval *δt* = *t*_*i*_ − *t*_*i*−1_, (*i* = 1, …, *m*) being 7 days for a total of *m* = 156 weeks constituting an analysis time-span of three years. Again following [7], we assumed a Poisson observation model for the number of new cases. Thus, the generative model is given by

$$\begin{array}{c} y_{t_{i}}∼\text{Poisson}\left(h_{\delta t}\left(C_{t}\right)\right),\;i=\left(1,\ldots ,m\right), \end{array}$$ (18)

where *h*_*δt*_(⋅) denotes the cumulative sum of *C*_*t*_ over the observation interval.

The parameters were set to the exact values used in [7] for generating the simulated data. We did the same for the initial values which were chosen in [7] to generate transients realistically reflecting a real application. To fit the data we employed the same SIRS model as in Eq (17), but we assumed the transmission-potential to evolve as an approximate Brownian motion, to capture a non-stationary signal, given by (following Eq (6))

$$\begin{array}{c} \frac{d\beta_{t}}{dt}=\sigma \sum_{i=1}^{n}Z_{i}\phi_{i}\left(t\right), \end{array}$$ (19)

where *σ* is a volatility parameter and we solve this ODE for *β*_*t*_ along with the compartmental ODEs in Eq (17), using an Euler solver. We chose *n* = 20, through a comparison of the marginal density at *p*(*β*_*t*_)|_*t*=*T*_, where *T* = 7 × 156 = 1092. We again chose the generalised Fourier basis in Eq (4) as the function *ϕ*_*i*_(*t*).

The unknown parameters included ***θ*** = (1/*α*, 1/*γ*, *σ*) (*μ* was assumed known) and the initial values ***X***_0_ = (*S*_0_, *I*_0_, *β*_0_). The Poisson observation model in Eq (18) was considered as the likelihood. We retained the same priors as in [7]. The priors and the generative values are given in Appendix J in S1 Text. We ran the adaptive MCMC algorithm, used previously for the influenza dataset, for 5 × 10^5^ iterations, discarded the first half as burn-in, and thinned appropriately to collect 1000 samples representing the posterior distribution. The run-time was **808** seconds, which is orders-of-magnitude faster than what was reported in [7].

**Fig 5:**
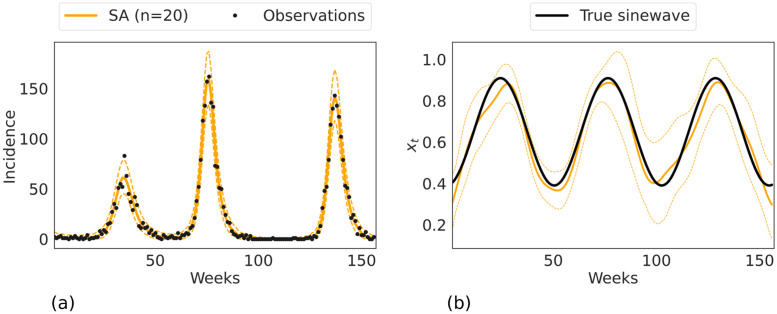
**SIRS model with sinusoidal transmission-potential**: Goodness-of-fit (a); posterior distribution of the latent diffusion paths corresponding to the **SDE** and **SA** approaches (b), with densities summarised by the mean (solid lines) and 95% credible intervals (broken lines).

In Fig 5 we plot the goodness-of-fit together with the posterior sample path of the transmission-potential. The posterior distribution of the parameters are shown in Appendix J in S1 Text. Note how (Fig 5(b)) the approximate Brownian motion model for *β*_*t*_ can recover well the true underlying signal, which clearly demonstrates that using an approximate diffusion process we can reliably reproduce the time evolution of the transmission-potential.

## 6 Application: Modelling COVID-19 outbreak in England

Our proposed method of modelling the time-varying transmission-potential as an approximate diffusion can also be applied to a discrete time stochastic process that converges to a diffusion in the continuous time limit. For example, an AR(1) process converges to a OU diffusion. Thus, if one is already using an AR(1) process to model the transmission-potential, then a discretised version of the series approximation of OU diffusion, the ODE in Eq (14), can be chosen as its replacement.

To exemplify the application of the series expansion method in replacing a discrete time stochastic process, we have chosen to fit a compartmental model, originally proposed in [8], whose dynamics are described as a set of first order difference equations, to data from the first wave of the COVID-19 outbreak in England, between February and August 2020. This model captures the effect of unknown extrinsic factors on the force of infection through a time-varying transmission-potential modelled as a Gaussian random-walk. We introduce the model of [8] in what follows and introduce an alternative formulation using the series approximation of Brownian motion.

### 6.1 Transmission model for COVID-19

This is an age and spatially structured transmission model, stratifying the population into *n*_*A*_ = 7 age groups and *n*_*r*_ = 7 regions. Within each region, the transmission dynamics are governed by a system of first order difference equations:

$$\begin{array}{c} \begin{array}{cc} S_{r,t_{k},a} & =S_{r,t_{k-1},a}(1-\lambda_{r,t_{k-1},a}\delta t)\\ E_{r,t_{k},a}^{1} & =E_{r,t_{k-1},a}^{1}(1-\frac{2\delta t}{d_{L}})+S_{r,t_{k-1},a}\lambda_{r,t_{k-1},a}\delta t\\ E_{r,t_{k},a}^{2} & =E_{r,t_{k-1},a}^{2}(1-\frac{2\delta t}{d_{L}})+E_{r,t_{k-1},a}^{1}\frac{2\delta t}{d_{L}}\\ I_{r,t_{k},a}^{1} & =I_{r,t_{k-1},a}^{1}(1-\frac{2\delta t}{d_{I}})+E_{r,t_{k-1},a}^{2}\frac{2\delta t}{d_{L}}\\ I_{r,t_{k},a}^{2} & =I_{r,t_{k-1},a}^{2}(1-\frac{2\delta t}{d_{I}})+I_{r,t_{k-1},a}^{1}\frac{2\delta t}{d_{I}} \end{array} \end{array}$$ (20)

where: $S_{r,t_{k},a}$, $E_{r,t_{k},a}^{d}$, $I_{r,t_{k},a}^{d},d=1,2$ represent the time *t*_*k*_, *k* = 1, …, *K*, partitioning of the population of individuals in a region *r*, *r* = 1, …, *n*_*r*_, in age-group *a*, *a* = 1, …, *n*_*A*_, into *S* (susceptible), *E* (exposed) and *I* (infectious) disease states. The average period spent in the exposed and infectious states are given by the parameters *d*_*L*_ and *d*_*I*_ respectively; and $\lambda_{r,t_{k},i}$ is the time- and age-varying rate with which susceptible individuals become infected, the force of infection. Time steps of *δt* = 0.5 days are chosen to be sufficiently small relative to the latent and infectious periods. Following [31] the initial conditions of the system states *S*, *E*^1^, *E*^2^, *I*^1^, *I*^2^ at *t*_0_ are given by region-specific parameters *ψ*_*r*_ and *I*_0,*r*_, describing the initial exponential growth and the initial number of infectious individuals, respectively. New infections are generated as

$$\begin{array}{c} \Delta_{r,t_{k},a}^{\text{infec}}=S_{r,t_{k},a}\lambda_{r,t_{k},a}\delta t, \end{array}$$ (21)

where $\lambda_{r,t_{k},a}\delta t$ is driven over time by a region-specific time-varying transmission potential, $\beta_{t_{k},r}$, (see Eq (22)) which moderates the rate at which effective contact take place. This region-specific transmission-potential captures the discrepancy between how actual contact take place between the age groups, and that encoded by a set of time-varying contact matrices. We refer the reader to [8] for further details on the model dynamics and parameterisation.

Over time $\beta_{t_{k},r}$ is not allowed to vary unconstrained and a smoothing is imposed by assuming, *a priori* that its evolution follows a Gaussian random-walk process with volatility $\sigma_{\beta_{t}}$:

$$\begin{array}{c} \begin{array}{cc} \text{log}(\beta_{t_{k},r}) & ∼N(\text{log}(\beta_{t_{k-1},r}),\sigma_{\beta_{t}}^{2}),\;\text{if}\;t_{k}>t_{\text{lock}},\\ \text{log}(\beta_{t_{k},r}) & =0,\;\text{if}\;t_{k}\leq t_{\text{lock}}, \end{array} \end{array}$$ (22)

where *t*_lock_ indicates the time-point corresponding to the lockdown introduced in England on 23^rd^ March 2020. This random-walk formulation requires the inference of the high-dimensional (due to the choice of *δt*) sample path of this process, an extremely challenging task using MCMC. We will discuss this inferential difficulty later in Section 6.3.2. To restrict the dimensionality of the process, the model formulation in [8] considered the transmission-potential to be piecewise constant with weekly changepoints, and its values at these changepoints to evolve following a random-walk. Denote *w*_*k*_ ≡ *w*(*t*_*k*_) the week in which time *t*_*k*_ falls. Then the time evolution of the transmission-potential is modelled at a slower weekly time-scale:

$$\begin{array}{c} \begin{array}{cc} \text{log}(\beta_{w_{k},r}) & ∼N(\text{log}(\beta_{w_{k-1},r}),\sigma_{\beta_{w}}^{2}),\;\text{if}\;t_{k}>t_{\text{lock}}+7/\delta t,\\ \text{log}(\beta_{w_{k},r}) & =0,\;\text{if}\;t_{k}\leq t_{\text{lock}}+7/\delta t,\\ \beta_{t_{k},r} & =\beta_{w_{k},r}, \end{array} \end{array}$$ (23)

as a Gaussian random-walk, with volatility $\sigma_{\beta_{w}}$, following the first week of the lockdown. Realisation of the process, for each region, can then be obtained by sampling the vector Δ***β***_*r*_ of all the weekly increments $\Delta \beta_{w_{k},r}=\text{log}(\beta_{w_{k},r})-\text{log}(\beta_{w_{k-1},r})$. It was assumed in [8] that the contact matrices sufficiently described how actual contacts took place between different age groups prior to the lockdown and thus $\beta_{w_{k},r}=1$ over that period. We have retained this assumption in our work.

### 6.2 Observation model

To fit the model, using a Bayesian framework, surveillance data of age- and region-specific counts of deaths in people with a lab-confirmed COVID-19 diagnosis between 17^st^ February and 1^st^ August was used. Furthermore, serological data from NHS Blood and Transplant (NHSBT), informing the fraction of the population carrying COVID-19 antibodies, were also used.

Following [8], the observed number of deaths $y_{r,t_{k},a}^{d}$ on day *t*_*k*_, in age group *a* in region *r* is the realisation of Negative Binomial distribution:

$$\begin{array}{c} y_{r,t_{k},a}^{d}|d_{I},p_{a},\psi_{r},I_{0_{r}},\Delta \beta_{r},\eta ∼\text{NegBin}(\mu_{r,t_{k},a},\eta ), \end{array}$$ (24)

where $Ey_{r,t_{k},a}^{d}=\mu_{r,t_{k},a}$ and $\text{Var}(y_{r,t_{k},a}^{d})=\mu_{r,t_{k},a}(1+\eta )$. Here *η* is a dispersion parameter and $\mu_{r,t_{k},a}=p_{a}\sum_{l=0}^{k}f_{k-l}\Delta_{r,t_{l},a}^{\text{infec}}$, with $\Delta_{r,t_{l},a}^{\text{infec}}$ obtained by solving the difference equations in Eq (20) and then evaluating Eq (21); *f*_*l*_ giving the probability of the death occurring on the *l*-th day after infection, assuming the distribution *f* of the time from infection to death from COVID-19 is known; and *p*_*a*_ representing an age-specific infection-fatality ratio.

The serological data are dependent on two parameters, the sensitivity and the specificity of the serological testing process, *k*_sens_ and *k*_spec_ respectively. If, on day *t*_*k*_, $n_{r,t_{k},a}$ blood samples are taken from individuals in region *r* and age-group *a*, and the observed number of positive tests is $y_{r,t_{k},a}^{s}$, then we assume following [8],

$$\begin{array}{c} y_{r,t_{k},a}^{s}|d_{I},\psi_{r},I_{0_{r}},\Delta \beta_{r},k_{\text{sens}},k_{\text{spec}}∼\text{Bin}(n_{r,t_{k},a},k_{\text{sens}}(1-\frac{S_{r,t_{k},a}}{N_{r,a}})+(1-k_{\text{spec}})\frac{S_{r,t_{k},a}}{N_{r,a}}), \end{array}$$ (25)

where $S_{r,t_{k},a}$ is obtained by solving the difference equations in Eq (20). *N*_*r*,*a*_ is the total population in age-group *a* and region *r*.

### 6.3 Inference

The unknown quantities that need to be inferred can be divided into two groups: (i) Global parameters $\theta_{g}=\left(\eta ,d_{I},p_{1},\ldots ,p_{n_{A}},k_{\text{sens}},k_{\text{spec}},\sigma_{\beta_{w}}\right)$ shared between regions, and (ii) regional parameters specific to each region: $\theta_{r}=\left(\psi_{r},I_{0_{r}},\Delta \beta_{r}\right)$. After placing the same priors as was used in [8] (and listed in Appendix E in S1 Text), the posterior distribution of the unknown quantities is as follows:

$$\begin{array}{c} p\left(\theta_{g},\theta_{1},\ldots ,\theta_{n_{r}}|y^{d},y^{s}\right)\propto p\left(\theta_{g}\right)\prod_{k=1}^{K}\prod_{a=1}^{n_{A}}\prod_{r=1}^{n_{r}}p\left(y_{r,t_{k},a}^{d}|\theta_{g},\theta_{r}\right)p\left(y_{r,t_{k},a}^{s}|\theta_{g},\theta_{r}\right)p\left(\theta_{r}\right), \end{array}$$ (26)

where we denote by $y^{d},y^{s}\in R^{K\times n_{A}\times n_{r}}$ the data for all time-points, ages and regions corresponding to deaths and sero-positive tests, respectively.

#### 6.3.1 Sampling from the posterior

Sampling from the posterior distribution Eq (26) is challenging due to the large number of random-walk increments corresponding to all regions and weeks since lockdown. MCMC with a vanilla RWMH proposal, as applied in [8], due to the linear scaling of convergence time with increasing dimensions mixes poorly and requires a large number of iterations (≈ 10^7^) of the Markov chain before convergence is reached. To improve convergence we instead used a random-scan Metropolis-within-Gibbs [32] (MwG) algorithm that circumvent the updating of a large parameter vector at each iteration. This MwG algorithm exploits the independence between the regional parameters. Our proposed sampling strategy consists of sampling alternatively, at each MCMC iteration, from the posterior of the global parameters conditioned on all the regional ones: (i) $p(\theta_{g}|\theta_{1},\ldots ,\theta_{n_{r}},y^{d},y^{s})$, and regional parameters for one randomly chosen region conditioned on the global ones (since the regional parameters are conditionally independent of any other region’s parameters): (ii) *p*(***θ***_r*_|***θ***_*g*_, ***y*^*d*^**, ***y*^*s*^**), where r* ∼ Uniform(1, *n*_*r*_). Samples from each of these conditional distributions are obtained using an adaptive RWMH move with the same adaptation mechanism used in Section 4.1. The pseudocode for this MwG algorithm is furnished in Appendix F in S1 Text.

#### 6.3.2 An alternative formulation

The number of region-specific random-walk increments $\Delta \beta_{w_{k},r}$ that needs to be sampled increases with time. The performance of the MwG algorithm starts deteriorating and exhibiting poor mixing and slow convergence, as this number becomes large. This limits dramatically the usefulness of this model in the context of a real-time application.

For the model in Eq (23), this problem can be tackled by increasing the time between two successive changepoints thus reducing the number of increments to be sampled for a period of analysis. This is however driven by computational convenience, and it would be more meaningful to learn these changes from data. We could model the time evolution of the transmission-potential at a faster time-scale, for example as in Eq (22). However, in this case the number of random-walk increments, to be sampled per region, equals the number of time-points between lockdown and the end of analysis date. Any MCMC sampler, that uses a RWMH proposal, would struggle severely to move efficiently in such a high-dimensional parameter space.

To alleviate these problems we propose to model the transmission-potential as a Brownian motion *W*_*t*,*r*_ with volatility $\sigma_{\beta_{t}}$ evolving in continuous time *t* and apply the series approximation as follows:

$$\begin{array}{cc} \beta_{t,r} & =\sigma_{\beta_{t}}\sum_{i=1}^{n}Z_{i,r}\int_{0}^{t}\phi_{i}(u)du\\ & =\sigma_{\beta_{t}}\sqrt{2T}\sum_{i=1}^{n}Z_{i,r}\frac{\text{sin}\{(i-1/2)\pi t/T\}}{(i-1/2)\pi }\approx W_{t}, \end{array}$$ (27)

where the second equality follows from choosing *ϕ*_*i*_ as given in Eq (4) and carrying out the integration. We can then discretise this approximation using the same time-step of *δt* that is used for the compartmental dynamics to obtain the following path-wise approximation evolving in discrete time *t*_*k*_:

$$\beta_{t_{k},r}=\sigma_{\beta_{t}}\sqrt{2T}\sum_{i=1}^{n}Z_{i,r}\frac{\text{sin}\{(i-1/2)\pi t_{k}/T\}}{(i-1/2)\pi },$$ (28)

where *T* is the number of days between lockdown and analysis date. Note that in this formulation the problem of sampling a large vector of increments Δ***β***_*r*_ is reduced to that of sampling a *n*-dimensional vector of the coefficients ***Z***_*r*_ = (*Z*_1,*r*_, …, *Z*_*n*,*r*_). From the comparison of the time *T* marginal distributions of the true and approximate Brownian motion, as for the OU process (see Fig 1), we found *n* = 10 to produce a good path-wise approximation. Thus, we used *n* = 10 for the subsequent comparative evaluations. The regional parameter vector, $\theta_{r}=\left(\psi_{r},I_{0_{r}},Z_{r}\right)$, now contains the expansion coefficients instead of the random-walk increments Δ***β***_*r*_.

### 6.4 Results: Comparative evaluations

We ran the MwG algorithm to target the posterior distribution in Eq (26) while using the random-walk based piecewise constant transmission-potential in Eq (23) and the Brownian motion approximation using series expansion with discretisation (SAd) in Eq (28). In both cases we ran 3 × 10^6^ iterations, discarded the first half of the iterations as burn-in and subsequently thinned the remaining samples to obtain 1000 samples. We implemented the epidemic model in C++. The MwG algorithm was implemented using Python.

**Fig 6:**
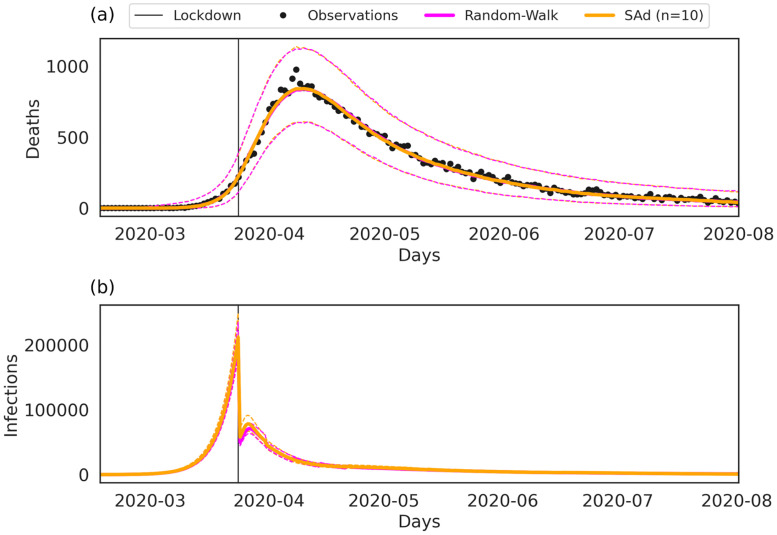
Goodness-of-fit of daily death data (a) and the inferred latent infections (b), produced using the random-walk (magenta lines) and SAd (orange lines). These densities are summarised by the mean (solid lines) and 95% credible intervals (broken lines). The black line indicates the day of lockdown in England 23^rd^ March, 2020.

Fig 6(a) compares, for the two alternative choices of modelling the transmission-potential, the posterior predictive distributions of the death data aggregated across all ages and regions with the observed data (see Appendix G in S1 Text for region-specific plots). Clearly the goodness-of-fit is indistinguishable between the two models. In Fig 6(b) we show summaries of the posterior distributions of the latent infections *p*(**Δ**^infec^|***y*^*d*^**, ***y*^*s*^**), aggregated across all ages and regions (region-wise infections are shown in Appendix G in S1 Text) again showing close consistency across models, with the exception of a few days immediately following the lockdown where the number of infections estimated by the SAd is slightly higher.

**Fig 7:**
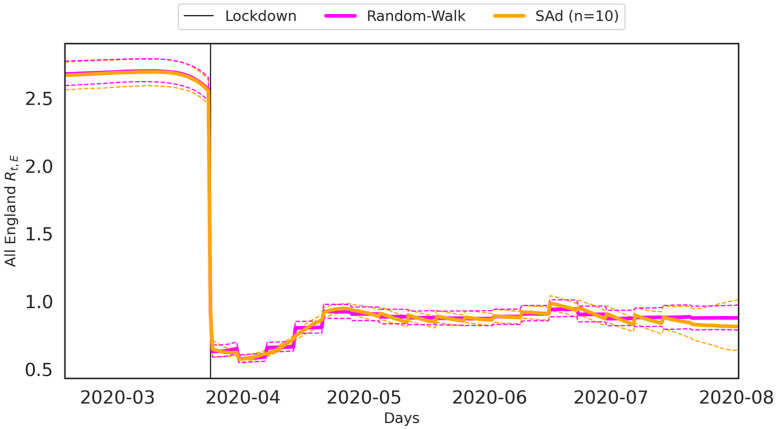
Posterior mean (solid lines) and 95% credible intervals (broken lines) for the all England reproduction number *R*_*t*,*E*_.

Following [8], we also obtain estimates of the effective region-specific reproduction number $R_{t_{k},r}$, their weighted average *R*_*t*,*E*_ representing the reproduction number for all of England, (formulae for these are given in Appendix D in S1 Text). In Fig 7 we show the posterior distributions for *p*(*R*_*t*,*E*_|***y*^*d*^**, ***y*^*s*^**), using the two alternative models. It is evident that the estimate obtained from the SAd appears to be smoother than what is obtained using the piecewise constant model, more realistically reflecting the actual transmission process that happens in continuous time. In Table 2 we present infection-fatality ratio estimates from the two models, again showing close agreement across models.

**Table 2:** Posterior mean and 95% credible intervals for the age-specific infection-fatality ratio from the random-walk and SAd models of transmission-potential.

Age group (yrs)	Random-walk	SAd
<5	0.00091% (0.00020%–0.0022%)	0.00093% (0.000071%–0.0019%)
15–14	0.0014% (0.00081%–0.0022%)	0.0014% (0.00062%–0.0022%)
15–24	0.0046% (0.0032%–0.0062%)	0.0044% (0.0029%–0.0060%)
25–44	0.031% (0.028%–0.034%)	0.029% (0.025%–0.034%)
45–64	0.46% (0.44%–0.49%)	0.44% (0.401%–0.49%)
65–74	3.0% (2.9%–3.2%)	2.9% (2.6%–3.3%)
> 74	17% (16%–18%)	17% (15%–18%)

#### Computational gains

The MwG algorithm took around 78 hours to finish for both models of the transmission-potential. However, the SAd allows inference at a faster time-scale producing a smoother estimate of *R*_*t*,*E*_ avoiding artificial model assumptions. Such an inference would be computationally infeasible if using a random-walk model at the more granular time-scale as in Eq (22), given the poor scaling of the RWMH proposal in high dimensions. Thus, using the series approximation we were able to extract more information about the transmission-potential and reproduction-ratio in comparison to the piecewise constant model, while incurring the same computational expense.

Had we used the random-walk model in Eq (22), we would have had to further partition each of the regional parameter block in separate chunks to accommodate a large vector of increments $\Delta \beta_{t_{k},r}=\text{log}(\beta_{t_{k},r})-\text{log}(\beta_{t_{k-1},r})$. Consequently, multiple Gibbs moves would have been necessary to update all the increments for a randomly chosen region. This, in turn, would have increased the number of likelihood computations, involving the computationally expensive updates of the transmission model, exponentially at each MCMC iteration.

## 7 Discussion

By modelling the force of infection as the function of a time-varying transmission-potential we can incorporate extrinsic, un-modelled effects in the description of the transmission process within a compartmental model. Describing this transmission-potential, in turn, as a stochastic process, a diffusion in particular, we can inject environmental stochasticity in an otherwise deterministic model. In this paper we proposed a path-wise approximation of a diffusion process as an alternative to modelling the dynamics of the transmission-potential as a SDE. Through the path-wise approximation we arrive at a random ODE approximating the SDE. As a function of its parameters, the path (solution) of an ODE is completely deterministic. As a result inference of the transmission-potential is simplified, with no need to solve a missing data problem using a computationally expensive data-augmentation procedure.

We demonstrate the efficacy of the proposed path-wise approximation using two epidemic models. In the first one, an influenza model, we replaced an OU SDE with an equivalent path-wise approximation. We noticed similar inference outcomes in terms of parameter estimates and goodness-of-fit using the SDE and its ODE approximation. In the second one, a toy SIRS model, we used an approximation of Brownian motion to reconstruct the non-stationary time evolution of a deterministic sinusoidal transmission potential from simulated data. The path-wise series approximation of Brownian motion was able to faithfully reconstruct the generative values of the sinusoidal transmission potential, as well as the model parameters. Importantly, for both models we observed orders-of-magnitude improvement in computational efficiency of the inference process when using a path-wise approximation of a diffusion process instead of the true diffusion.

We then applied the path-wise approximation to replace a Gaussian random-walk with a discretised path-wise approximation of Brownian motion to model the transmission-potential within a compartmental model of COVID-19 pandemic spread in England. Again we noticed consistent estimates of crucial unknown quantities such as infection-fatality rate, latent infections and a time-varying estimate of the reproduction number. In addition, the path-wise approximation allows the transmission-potential to be modelled at a more granular time-scale providing a smooth estimate of the effective reproduction number. This would be impossible to achieve using the random-walk model due to an exorbitant computational burden.

As an alternative to using our path-wise approximation of Brownian motion to model the transmission-potential, at a faster time-scale, we could have used a different MCMC algorithm, such as the No-U-Turn sampler [33], that is known to perform well for high dimensional problems. This algorithm proposes a move based on the gradient of the target density. Evaluating gradients, however, for the COVID-19 model is challenging as this requires, in addition to extra computations, a complete re-implementation of the model using an automatic differentiation package. However, for modelling studies where such re-implementation is straightforward, we like to point out that by applying a path-wise approximation of a diffusion process we are left with the task of sampling from a posterior distribution with a standard Gaussian prior (over the coefficients). The No-U-Turn sampler generally excels at this task.

In this paper we have used simple diffusion models whose transition densities are known analytically. However, if additional prior information about the force of infection is available, then such information can be incorporated in more complex nonlinear SDEs as models of the time-varying transmission-potential. Moreover, SDEs can be used to model any other time-varying parameters, in addition to the transmission-potential. Our methodology can be seamlessly applied in all such cases to arrive at a path-wise approximation of such complex diffusion processes.

Any time-varying parameter, that is modelled as a diffusion process, can also be modelled using our proposed approximate diffusion process. The usefulness or necessity of considering multiple time-varying parameters, beyond just the transmission potential, depends on the particular application context. In this work our focus primarily was to show the speed-up in inference that can be achieved using an approximate diffusion model of the transmission potential only.

## Supporting information

S1 Text Contains a derivation of the Fourier expansion of Brownian motion (Appendix A), description of the adaptation of the RWMH proposal (Appendix B), simulation study mimicking the influenza dataset (Appendix C), formulae for the reproduction number (Appendix D), priors for the COVID-19 model parameters (Appendix E), pseudocode of the MwG algorithm (Appendix F), region-specific plots for the COVID-19 model (Appendix G), description of the MMD metric (Appendix H), additional experiment using the influenza dataset (Appendix I) and further details of the SIRS model (Appendix J).
(PDF)
