# Supplementary material for: An approximate diffusion process for environmental stochasticity in infectious disease transmission modelling
Source: PLoS Comput Biol. 2023 May 18;19(5):e1011088. doi: 10.1371/journal.pcbi.1011088 (PMC10231796; doi:10.1371/journal.pcbi.1011088)
Supplement: S1 Text — Contains a derivation of the Fourier expansion of Brownian motion (Appendix A), description of the adaptation of the RWMH proposal (Appendix B), simulation study mimicking the influenza dataset (Appendix C), formulae for the reproduction number (Appendix D), priors for the COVID-19 model parameters (Appendix E), pseudocode of the MwG algorithm (Appendix F), region-specific plots for the COVID-19 model (Appendix G), description of the MMD metric (Appendix H), additional experiment using the influenza dataset (Appendix I) and further details of the SIRS model (Appendix J). (PDF) [file pcbi.1011088.s001.pdf]

Supplementary Materials: An approximate diffusion process for environmental stochasticity in infectious disease transmission modelling

Sanmitra Ghosh [1], Paul J. Birrell[2,1], Daniela De Angelis[1,2]
[1] *MRC Biostatistics Unit, University of Cambridge, Cambridge, UK*
[2] *UK Health Security Agency, London, UK*

Appendix A: Fourier expansion of Brownian motion

By the definition of an Itô integral, within a time interval $[0, T]$ a standard Brownian motion can be written as [1, 2]:

$$W_t = \int_0^t dW_s = \int_0^T \mathbb{I}_{[0,t]}(s) dW_s, \tag{1}$$

where $\mathbb{I}_{[0,t]}(\cdot)$ is the indicator function. Suppose $\{\phi_i\}_{i=1}^{\infty}$ is a complete orthonormal basis of $L^2[0, T]$. We can interpret $\mathbb{I}_{[0,t]}$ as an element of $L^2[0, T]$, and expand it in terms of the basis functions:

$$\begin{aligned} \mathbb{I}_{[0,t]}(s) &= \sum_{i=1}^{\infty} \langle \mathbb{I}_{[0,t]}(\cdot), \phi_i(\cdot) \rangle \phi_i(s) \\ &= \sum_{i=1}^{\infty} \Big(\int_0^t \phi_i(u) du \Big) \phi_i(s). \end{aligned} \tag{2}$$

Substituting (2) into (1) we see that:

$$W_t = \sum_{i=1}^{\infty} \Big(\int_0^T \phi_i(s) dW_s \Big) \int_0^t \phi_i(u) du. \tag{3}$$

Appendix B: Adaptive MCMC

In an adaptive MCMC algorithm optimal values of the proposal density is learnt on the fly using past samples from the Markov chain. Different mechanisms can be used to adapt or learn the parameters of the proposal. [3] proposed a general framework for constructing adaptive MCMC algorithms that rely on the *stochastic approximation* method [4] for learning the proposal's parameters on the fly.

Consider in general the proposal density $q_\phi(\boldsymbol{\theta}^{j+1}|\boldsymbol{\theta}^j)$ parameterised by ϕ. Let us also define a suitable objective function

$$h(\phi) := \mathbb{E}^{\phi}\big[H(\phi, \boldsymbol{\theta}^0, \boldsymbol{\theta}^1, \ldots, \boldsymbol{\theta}^j, \boldsymbol{\theta}^{j+1})\big], \tag{4}$$

that expresses some measure of the statistical performance of the Markov chain in its stationary regime. The expectation is with respect to a ϕ dependent distribution. For example, the coerced

acceptance probability is often used as the objective:

$$H(\phi, \boldsymbol{\theta}^0, \boldsymbol{\theta}^1, \dots, \boldsymbol{\theta}^j, \boldsymbol{\theta}^{j+1}) = \underbrace{\min\left\{1, \frac{\pi(\boldsymbol{\theta}^{j+1})}{\pi(\boldsymbol{\theta}^j)} \frac{q_\phi(\boldsymbol{\theta}^j|\boldsymbol{\theta}^{j+1})}{q\phi(\boldsymbol{\theta}^{j+1}|\boldsymbol{\theta}^j)}\right\}}_{=:\alpha^{j+1}} - \bar{\alpha}, \tag{5}$$

where $\pi(\boldsymbol{\theta})$ is the target distribution and $\bar{\alpha}$ is the approximate optimal expected acceptance probability in the stationary regime. For the Gaussian proposal $q := \mathcal{N}(\boldsymbol{\theta}^{j+1}|\boldsymbol{\theta}^j, \boldsymbol{\Sigma}^j)$, with its parameter ϕ being the covariance $\boldsymbol{\Sigma}^j$, the following objective function:

$$H(\boldsymbol{\Sigma}^j, \boldsymbol{\theta}^{j+1}) = \boldsymbol{\theta}^{j+1}\boldsymbol{\theta}^{j+1'} - \boldsymbol{\Sigma}^j, \tag{6}$$

corresponds to matching the moments of the proposal with that of the target. Here by a' we denote the transpose of the vector a.

Optimal exploration of $\pi(\boldsymbol{\theta})$ can thus be formulated as finding the root $\bar{\phi}$ of the following equation: $h(\phi) = 0$. The challenge here is to devise an algorithm to find the roots of $h(\phi)$, which involves both integration and optimisation. [3] suggested using the stochastic approximation method [4] which is tailored to this situation:

$$\begin{aligned} \phi^{j+1} &= \phi^j + \delta^{j+1} H(\phi^j, \boldsymbol{\theta}^0, \boldsymbol{\theta}^1, \dots, \boldsymbol{\theta}^j, \boldsymbol{\theta}^{j+1}) \\ &= \phi^j + \delta^{j+1} h(\phi) + \delta^{j+1} H(\phi^j, \boldsymbol{\theta}^0, \boldsymbol{\theta}^1, \dots, \boldsymbol{\theta}^j, \boldsymbol{\theta}^{j+1}) - \delta^{j+1} h(\phi) \\ &= \phi^j + \delta^{j+1} h(\phi) + \delta^{j+1} \xi^{j+1}, \end{aligned} \tag{7}$$

where $\xi^{j+1} := \left[H(\phi^j, \boldsymbol{\theta}^0, \boldsymbol{\theta}^1, \dots, \boldsymbol{\theta}^j, \boldsymbol{\theta}^{j+1}) - h(\phi)\right]$ is usually referred to as the *noise term* and δ^j is a decreasing sequence (a step-size parameter). If the noise term ξ^{j+1} averages to zero as $j \to \infty$, the above recursion will converge to the root $\bar{\phi}$ (or at least oscillate around it) when the following conditions hold:

$$\sum_{j=0}^{\infty} \delta^j = \infty \quad \text{and} \quad \sum_{j=0}^{\infty} (\delta^j)^2 < \infty. \tag{8}$$

Combining the above objective functions and using the stochastic approximation we have the following recursions for adapting a random-walk proposal with a global scaling λ^j, $\mathcal{N}(\boldsymbol{\theta}^{j+1}|\boldsymbol{\theta}^j, \lambda^j\boldsymbol{\Sigma}^j)$, as [3]:

$$\begin{aligned} \log(\lambda^{j+1}) &= \log(\lambda^j) + \delta^{j+1}(\alpha^{j+1} - \bar{\alpha}) \\ \boldsymbol{\mu}^{j+1} &= \boldsymbol{\mu}^j + \delta^{j+1}(\boldsymbol{\theta}^{j+1} - \mu^j) \\ \boldsymbol{\Sigma}^{j+1} &= \boldsymbol{\Sigma}^j + \delta^{j+1}(\boldsymbol{\theta}^{j+1}\boldsymbol{\theta}^{j+1'} - \boldsymbol{\Sigma}^j), \end{aligned} \tag{9}$$

where the recursion in the first equation, trying to adapt the global scaling, is based on the coerced accepted probability objective in (5) and the following two equations are minimising the moment matching objective in (6).

By choosing a decreasing sequence $\{\delta^j\}_{j=0}^{\infty}$ of step-sizes it is ensured that the adaptation declines over time, also known as *vanishing adaptation* [3], and the Markov chain converges to the correct stationary distribution. For all the experiments we have consistently used the following schedule:

$$\delta^j = j^{-0.6}, \tag{10}$$

which was shown to work particularly well for nonlinear differential equation models in [5].

Appendix C: Simulation study for influenza epidemic

Using a real dataset we are oblivious to the ground truth of the estimated quantities. Thus, we have also carried out a detailed simulation study where we have used simulated datasets that mimic the influenza epidemic used in the main text. We generated three simulated epidemics using the model in Eq (2), in the main text, on the same time period $T = 14$ days, and used the same population size $N = 763$, as the real influenza epidemic. We chose parameter values that generate an epidemic curve similar to the real dataset. These generative parameter values are shown in Figure 1–3. We then proceed to fit the two alternative models using the inferential setup discussed in the main text.

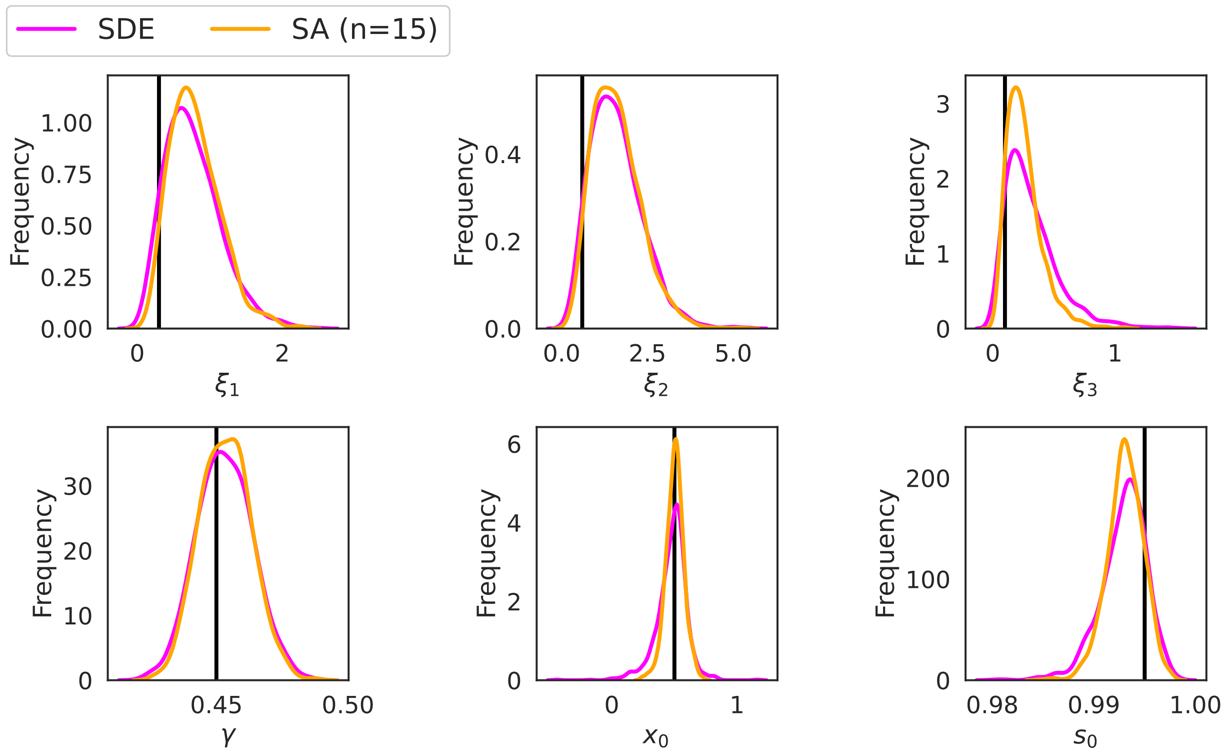

Figure 1: **Simulated dataset 1**: Posterior marginal densities of the parameters obtained using the **SDE** and the **SA** (with $n = 15$ basis function). These densities are summarised using a kernel density estimate. The black line in each of the plots indicate the generative parameter value.

In Figure 1–3 we compared the marginal densities of the parameters obtained using the **SDE** and **SA** counterparts, for each of the simulated datasets. Clearly the estimates match well and generative parameter values are recovered.

Furthermore, in Figure 4–6 we compared the goodness-of-fit. As was found for the real dataset, we observed little disagreement between the epidemic curves obtained using the **SDE** and the **SA**, but for the posterior distribution of the latent diffusion paths we noticed, for all the datasets, that the credible intervals are narrower for the **SA**. For all these datasets, the posterior means, and the draws of the sample path, of the two models match well.

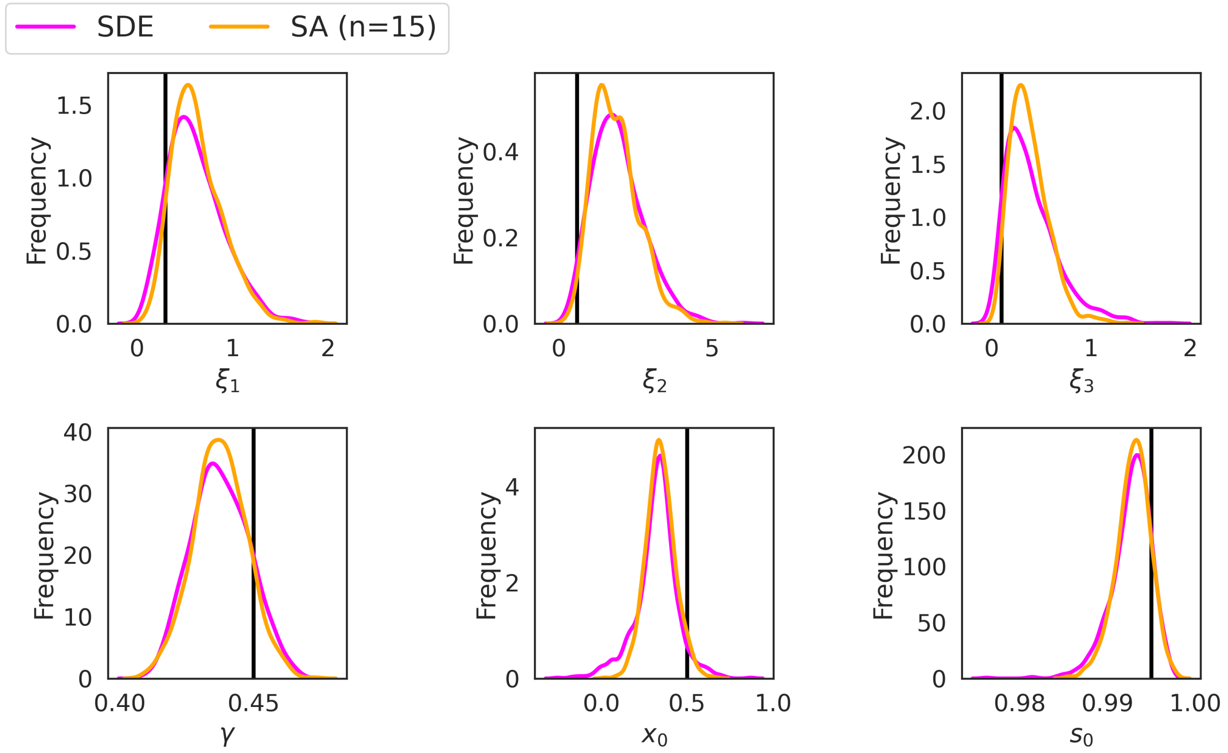

Figure 2: **Simulated dataset 2**: Posterior marginal densities.

Appendix D: Calculating a time-varying reproduction number

The estimate of the contact-rate $\beta_{t_k,r}$ is used to derive an estimate of a time-varying reproduction number. Firstly, using the formula of [6], the initial reproduction number $R_{0,r}$ is estimated as follows:

$$R_{0,r} = \psi_r d_I \frac{\left(\frac{\psi_r d_L}{2} + 1\right)^2}{1 - \frac{1}{\left(\frac{\psi_r d_I}{2}+1\right)^2}}. \tag{11}$$

Over time the value of the reproduction number will change as contact patterns shift and the supply of susceptible individuals deplete. The time-t reproduction number is then estimated using the following formula:

$$R_{t_k,r} = \begin{cases} R_{0,r} \frac{R^*_{t_k,r}}{R^*_{0,r}} & \text{if } t_k < t_{\text{lock}} \\ \beta_{t_k,r} R_{0,r} \frac{R^*_{t_k,r}}{R^*_{0,r}} & \text{if } t_k \geq t_{\text{lock}} \end{cases} \tag{12}$$

where t_{lock} indicates the time-point corresponding to the lockdown. $R^*_{t_k,r}$ is the dominant eigenvalue of the time t_k next-generation matrix, $\Lambda_{k,r}$, with elements:

$$(\Lambda_{k,r})_{ij} = S_{r,t_k,i} \boldsymbol{C}^{t_k}_{r,ij} d_I, \tag{13}$$

where $\boldsymbol{C}^{t_k}_{r,ij}$ is a region-specific time-varying contact matrix, see [7] for further details on these matrices.

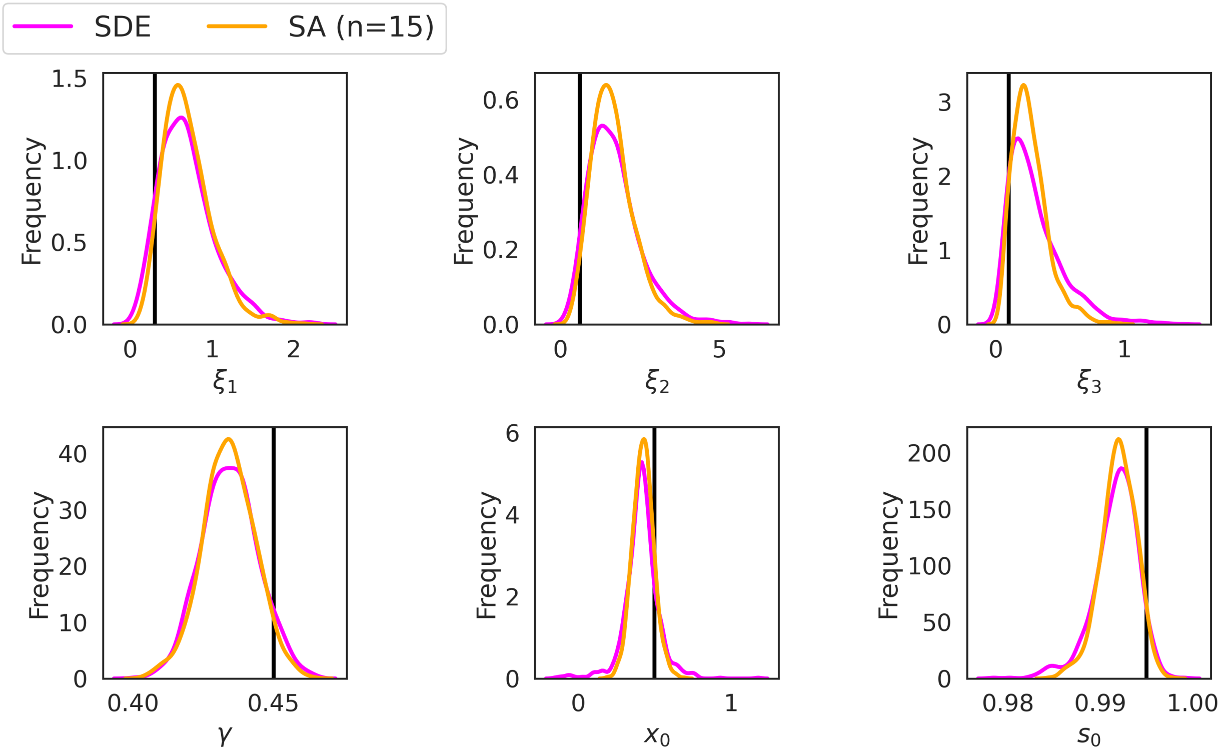

Figure 3: **Simulated dataset 3**: Posterior marginal densities.

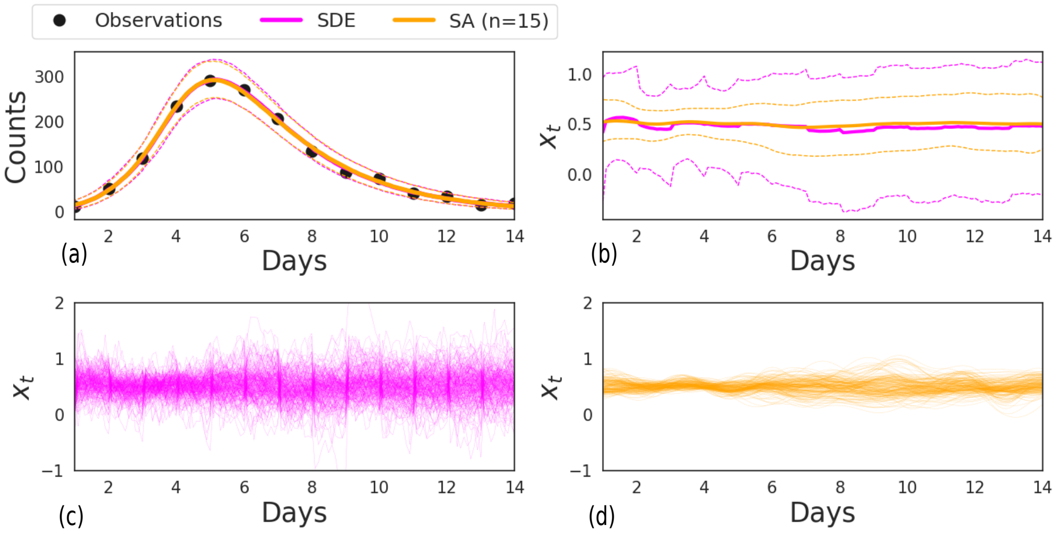

Figure 4: **Simulated dataset 1**: Goodness-of-fit (a); posterior distribution of the latent diffusion paths corresponding to the **SDE** and **SA** counterparts (b), with densities summarised by the mean (solid lines) and 95% credible intervals (broken lines); and samples from the posterior distribution of the latent diffusion paths, **SDE** (c) and **SA** (d)

To get an 'all England' value for $R_{t_k,E}$ a weighted average of the regional $R_{t_k,r}$ is calculated,

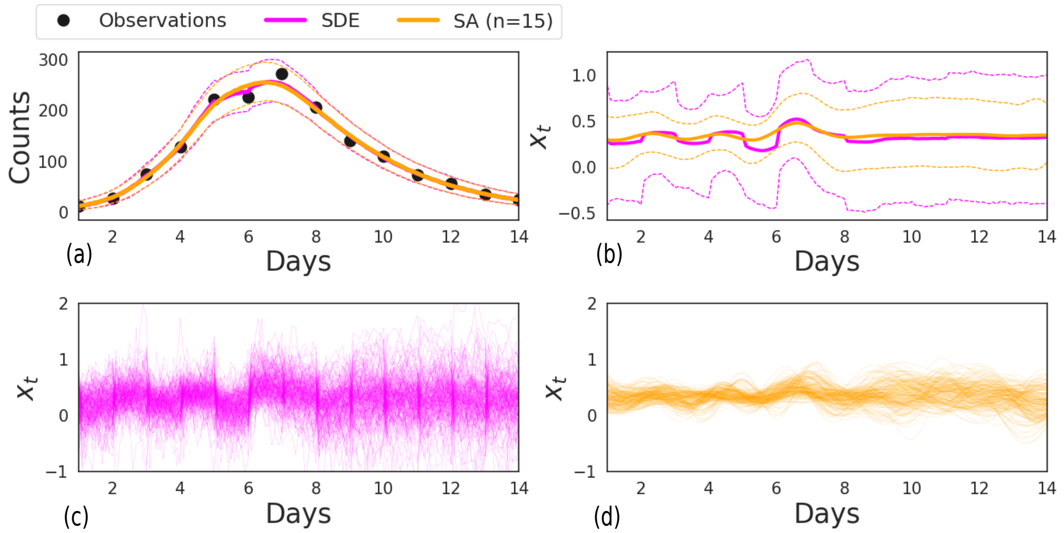

Figure 5: **Simulated dataset 2**: Comparison of the goodness-of-fit

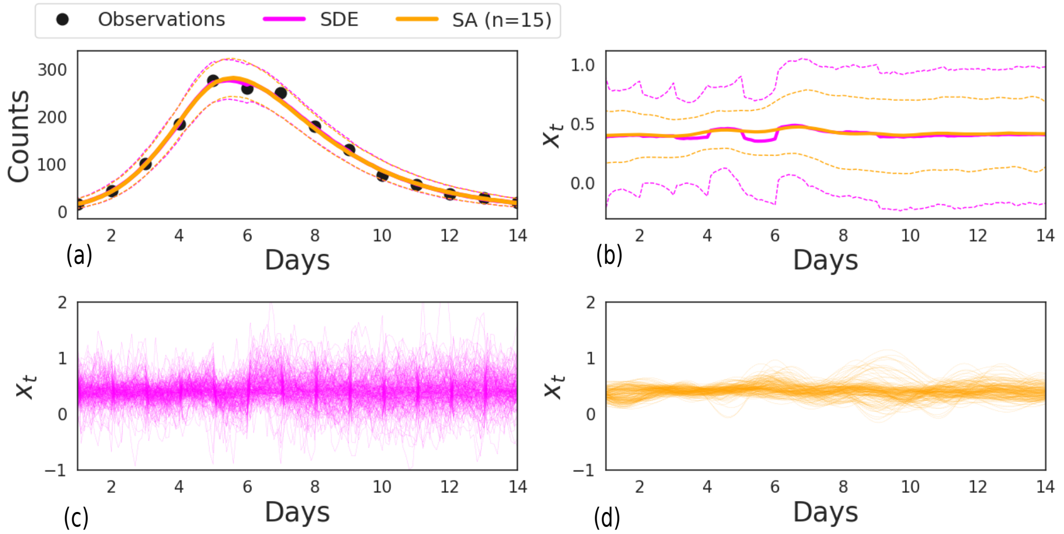

Figure 6: **Simulated dataset 3**: Comparison of the goodness-of-fit

where the weights are given by the sum of the infections in each region:

$$R_{t_k,E} = \frac{\sum_r R_{t_k,r} \sum_i \Delta^{\text{infec}}_{r,t_k,i}}{\sum_r \sum_i \Delta^{\text{infec}}_{r,t_k,i}}. \tag{14}$$

Appendix E: Priors for the COVID-19 model

The priors for the global and regional parameters for the COVID-19 model are listed in Table 1. We used the same priors as was used in [7]. Note that we also used the same prior for the volatility of both the piecewise constant random-walk and the Brownian motion model of the transmission-potential.

Table 1: Model parameters with assumed prior distributions or fixed values, as was used in [7].

Name	Prior source
Over-dispersion, η	Uninformative Gamma$(1, 0.2)$.
Mean infectious period, d_I	$2 +$ Gamma$(1.43, 0.549)$.
Infection-fatality rate for age < 5: p_1	Beta$(1, 62110.8012)$.
Infection-fatality rate for age, $5 - 14$: p_2	Beta$(1, 23363.4859)$.
Infection-fatality rate for age $15 - 24$: p_3	Beta$(1, 5290.0052)$.
Infection-fatality rate for age $25 - 44$: p_4	Beta$(1, 1107.6474)$.
Infection-fatality rate for age $45 - 64$: p_5	Beta$(1, 120.9512)$.
Infection-fatality rate for age $65 - 74$: p_6	Beta$(1, 31.1543)$.
Infection-fatality rate for age > 74: p_7	Beta$(9.5, 112)$.
Serological test sensitivity, k_{sens}	Beta$(71.5, 29.5)$.
Serological test specificity, k_{spec}	Beta$(777.5, 9.5)$.
Exponential growth, ψ_r	Gamma$(31.36, 224)$.
Log of initial infectives, $\log I_{0,r}$	$\mathcal{N}(-17.5, 1.25^2)$.
Volatility of transmission-potential, $\sigma_{\beta_w}, \sigma_{\beta_t}$	Gamma$(1, 100)$.
Mean latent period, d_L	3 days (fixed not estimated).

Appendix F: Pseudocode of the MwG algorithm

The pseudocode listed in Algorithm 1 describes the Metropolis-within-Gibbs algorithm for sampling from the posterior distribution $p(\boldsymbol{\theta}_g, \boldsymbol{\theta}_1, \ldots, \boldsymbol{\theta}_{n_r} | \boldsymbol{y^d}, \boldsymbol{y^s})$ of the global $\boldsymbol{\theta}_g$ and regional $\boldsymbol{\theta}_1, \ldots, \boldsymbol{\theta}_{n_r}$ parameters of the COVID-19 model. For each parameter group $\boldsymbol{\theta}_g, \boldsymbol{\theta}_1, \ldots, \boldsymbol{\theta}_{n_r}$ we use a proposal with a different set of parameters that are adapted through the mechanism described in (9).

Appendix G: Goodness-of-fit as per regions of England

In Figure 10 – 16 we show the posterior predictive distributions of the number of deaths and the posterior distribution of the latent infection for each region respectively. We have aggregated the results across ages.

Appendix H: Maximum mean discrepancy

For any given probability distribution $\mathbb{P}$ on a domain $\mathcal{X}$ its kernel embedding is defined as $\mu_{\mathbb{P}} = \mathbb{E}_{X\sim\mathbb{P}} k(\cdot, \boldsymbol{\theta})$ [8], an element of reproducing kernel Hilbert space $\mathcal{H}$ associated with a positive definite kernel function $k : \mathcal{X} \times \mathcal{X} \to \mathbb{R}$. Such an embedding exists for any $\mathbb{P}$ whenever k is bounded. Given two probability distributions $\mathbb{P}$ and $\mathbb{Q}$ the maximum mean discrepancy (MMD) is the Hilbert space distance between their kernel embedding $\mu_{\mathbb{P}}$ and $\mu_{\mathbb{Q}}$. Considering that we have two set of samples $\{X_i\}_{i=1}^n$ and $\{Y_i\}_{i=1}^m$ from corresponding distributions $\mathbb{P}$ and $\mathbb{Q}$ respectively, then the MMD between $\mathbb{P}$ and $\mathbb{Q}$ is given by [9]

$$\begin{aligned} MMD^2(\mathbb{P}, \mathbb{Q}) &= ||\mu_{\mathbb{P}} - \mu_{\mathbb{Q}}||_{\mathcal{H}} \\ &= \frac{1}{n(n-1)} \sum_{i=1}^{n} \sum_{j \neq i}^{m} k(X_i, X_j) + \frac{1}{m(m-1)} \sum_{i=1}^{n} \sum_{j \neq i}^{m} k(Y_i, Y_j) - \frac{2}{nm} \sum_{i=1}^{n} \sum_{j=1}^{m} k(X_i, Y_j). \end{aligned} \tag{15}$$

Algorithm 1 A random-scan adaptive Metropolis-within-Gibbs sampler

Input: Number of iterations J; data $\boldsymbol{y^d}, \boldsymbol{y^s}$; optimal acceptance rate $\bar{\alpha}$.
Initialise the regional $\boldsymbol{\theta}_1^0, \ldots, \boldsymbol{\theta}_{n_r}^0$ and global parameters $\boldsymbol{\theta}_g^0$.
Initialise the regional proposal parameters $\lambda_1^0, \ldots, \lambda_{n_r}^0$, $\boldsymbol{\mu}_1^0, \ldots, \boldsymbol{\mu}_{n_r}^0$ and $\boldsymbol{\Sigma}_1^0, \ldots, \boldsymbol{\Sigma}_{n_r}^0$.
Initialise the global proposal's parameters λ_g^0, $\boldsymbol{\mu}_g^0$ and $\boldsymbol{\Sigma}_g^0$.
for $j = 0$ **to** $J-1$ **do**

Global move:

1. Draw $\boldsymbol{\theta}_g^* \sim \mathcal{N}(\boldsymbol{\theta}_g^j, \lambda_g^j \boldsymbol{\Sigma}_g^j)$ and set $\boldsymbol{\theta}_g^{j+1} = \boldsymbol{\theta}_g^*$ with probability $\alpha_g^{j+1} = \min\left\{1, \frac{p(\boldsymbol{\theta}_g^* | \boldsymbol{y^d}, \boldsymbol{y^s})}{p(\boldsymbol{\theta}_g | \boldsymbol{y^d}, \boldsymbol{y^s})}\right\}$, otherwise $\boldsymbol{\theta}_g^{j+1} = \boldsymbol{\theta}_g^j$.

Regional move:

1. Draw $r^* \sim \text{Uniform}(1, n_r)$.
2. Draw $\boldsymbol{\theta}_{r^*}^* \sim \mathcal{N}(\boldsymbol{\theta}_{r^*}^j, \lambda_{r^*}^j \boldsymbol{\Sigma}_{r^*}^j)$ and set $\boldsymbol{\theta}_{r^*}^{j+1} = \boldsymbol{\theta}_{r^*}^*$ with probability $\alpha_{r^*}^{j+1} = \min\left\{1, \frac{p(\boldsymbol{\theta}_{r^*}^* | \boldsymbol{y^d}, \boldsymbol{y^s})}{p(\boldsymbol{\theta}_{r^*}^j | \boldsymbol{y^d}, \boldsymbol{y^s})}\right\}$, otherwise $\boldsymbol{\theta}_{r^*}^{j+1} = \boldsymbol{\theta}_{r^*}^j$.
3. Set $\boldsymbol{\theta}_{n_r \setminus r^*}^{j+1} = \boldsymbol{\theta}_{n_r \setminus r^*}^j$, where the symbol $A \setminus a$ denotes all elements of the set A except a.

Adaptation:

1. Adapt global proposal's parameters:

$$\begin{aligned}
\log(\lambda_g^{j+1}) &= \log(\lambda_g^j) + \delta^{j+1}(\alpha_g^{j+1} - \bar{\alpha}) \\
\boldsymbol{\mu}_g^{j+1} &= \boldsymbol{\mu}_g^j + \delta^{j+1}(\boldsymbol{\theta}_g^{j+1} - \boldsymbol{\mu}_g^j) \\
\boldsymbol{\Sigma}_g^{j+1} &= \boldsymbol{\Sigma}_g^j + \delta^{j+1}(\boldsymbol{\theta}_g^{j+1} \boldsymbol{\theta}_g^{j+1'} - \boldsymbol{\Sigma}_g^j).
\end{aligned}$$

2. Adapt proposal's parameters for region r^*:

$$\begin{aligned}
\log(\lambda_{r^*}^{j+1}) &= \log(\lambda_{r^*}^j) + \delta^{j+1}(\alpha_{r^*}^{j+1} - \bar{\alpha}) \\
\boldsymbol{\mu}_{r^*}^{j+1} &= \boldsymbol{\mu}_{r^*}^j + \delta^{j+1}(\boldsymbol{\theta}_{r^*}^{j+1} - \boldsymbol{\mu}_{r^*}^j) \\
\boldsymbol{\Sigma}_{r^*}^{j+1} &= \boldsymbol{\Sigma}_{r^*}^j + \delta^{j+1}(\boldsymbol{\theta}_{r^*}^{j+1} \boldsymbol{\theta}_{r^*}^{j+1'} - \boldsymbol{\Sigma}_{r^*}^j).
\end{aligned}$$

3. Set $\lambda_{n_r \setminus r^*}^{j+1} = \lambda_{n_r \setminus r^*}^j$, $\boldsymbol{\mu}_{n_r \setminus r^*}^{j+1} = \boldsymbol{\mu}_{n_r \setminus r^*}^j$ and $\boldsymbol{\Sigma}_{n_r \setminus r^*}^{j+1} = \boldsymbol{\Sigma}_{n_r \setminus r^*}^j$.

end for
Output: $\{\boldsymbol{\theta}_g^j, \boldsymbol{\theta}_1^j, \ldots, \boldsymbol{\theta}_{n_r}^j\}_{j=0}^{J-1}$.

The $MMD^2(\mathbb{P}, \mathbb{Q}) = 0$ iff $\mathbb{P} = \mathbb{Q}$, following the properties of kernel embedding. The kernel embedding captures all the necessary information about a distribution [8], thus the distance between two embedding would naturally highlight the discrepancy more efficiently in the tail regions of the distributions under comparison. In this paper we used an exponentiated quadratic kernel given by

$$k(X, X') = \exp\Big(\frac{||X - X'||^2}{\rho^2}\Big), \tag{16}$$

where ρ is a hyperparameter. We set ρ to the median distance among the samples.

Appendix I: Considering overdispersion while fitting the influenza dataset

In section 4 (main text) while fitting to the influenza dataset we considered a Poisson likelihood model, for both the **SA** and **SDE** variants, given by Eq (14) and Eq (15) respectively. Such a likelihood model formulation ignores overdispersion in the measurement process. Interestingly, not allowing for overdispersion in the measurement process may lead to the compensation of variability in the observations through spurious changes in the transmission-potential. We noticed in Figure 3 (b) (main text) that there is a noticeable increase in x_t, and thus transmission-potential. In order to rule out such increase as something spurious, we re-fit both the **SA** and **SDE** variants, in this section, using a negative binomial likelihood. More specifically, we consider the following likelihood models for the **SDE**:

$$y_{t_i}|\boldsymbol{\theta}, \boldsymbol{x}, \boldsymbol{X}_0 \sim \text{NegBin}(I_{t_i}, \eta), \quad i = 1, \dots, m, \tag{17}$$

where η is an overdispersion parameter such that $\mathbb{E}y_{t_i} = I_{t_i}$ and $\text{Var}\,(y_{t_i}) = I_{t_i}\,(1+\eta)$, and the **SA**:

$$y_{t_i}|\boldsymbol{\theta}, \boldsymbol{Z}, \boldsymbol{X}_0 \sim \text{NegBin}(I_{t_i}, \eta). \tag{18}$$

We retained all the experimental setting as was used in section 4 and carried out inference, while additionally estimating the overdispersion parameter η. We placed a Gamma$(2, 5)$ prior on η.

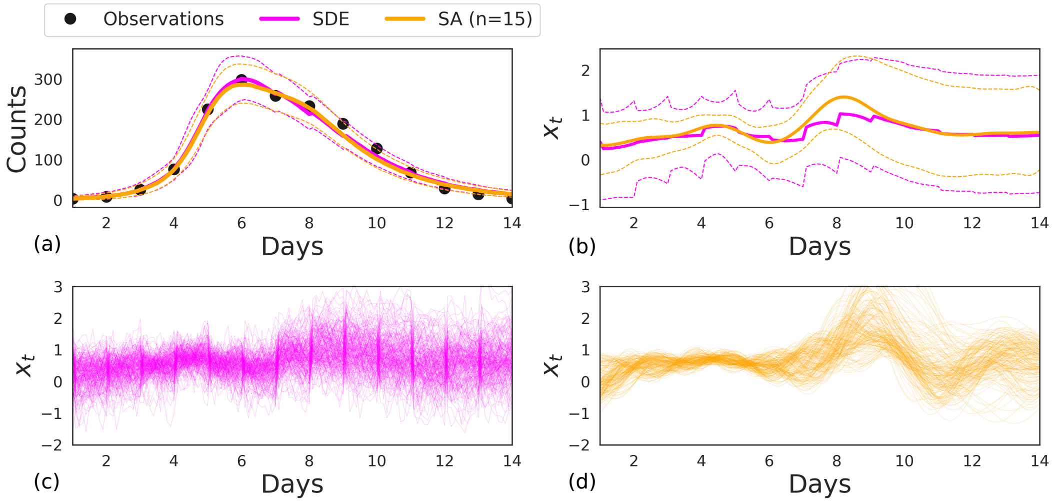

Figure 7: **Considering a negative binomial likelihood for fitting the influenza dataset**: Goodness-of-fit (a); posterior distribution of the latent diffusion paths corresponding to the **SDE** and **SA** counterparts (b), with densities summarised by the mean (solid lines) and 95% credible intervals (broken lines); and samples from the posterior distribution of the latent diffusion paths, **SDE** (c) and **SA** (d)

In Figure 7 we show the goodness-of-fit and the posterior distribution of the latent diffusion's sample path. In Figure 8 we plot the posterior distribution of the parameters. The main difference that we noticed, in comparison to the Poisson likelihood model, is that the Poisson likelihood model produced a closer fit (see Figure 3 (a) in main text) to the observation on the 9-th day, for both the **SA** and **SDE** variants. Importantly, both the Poisson and negative binomial likelihood models picked-up the increase in the transmission-potential between the 6-th and the 9-th day.

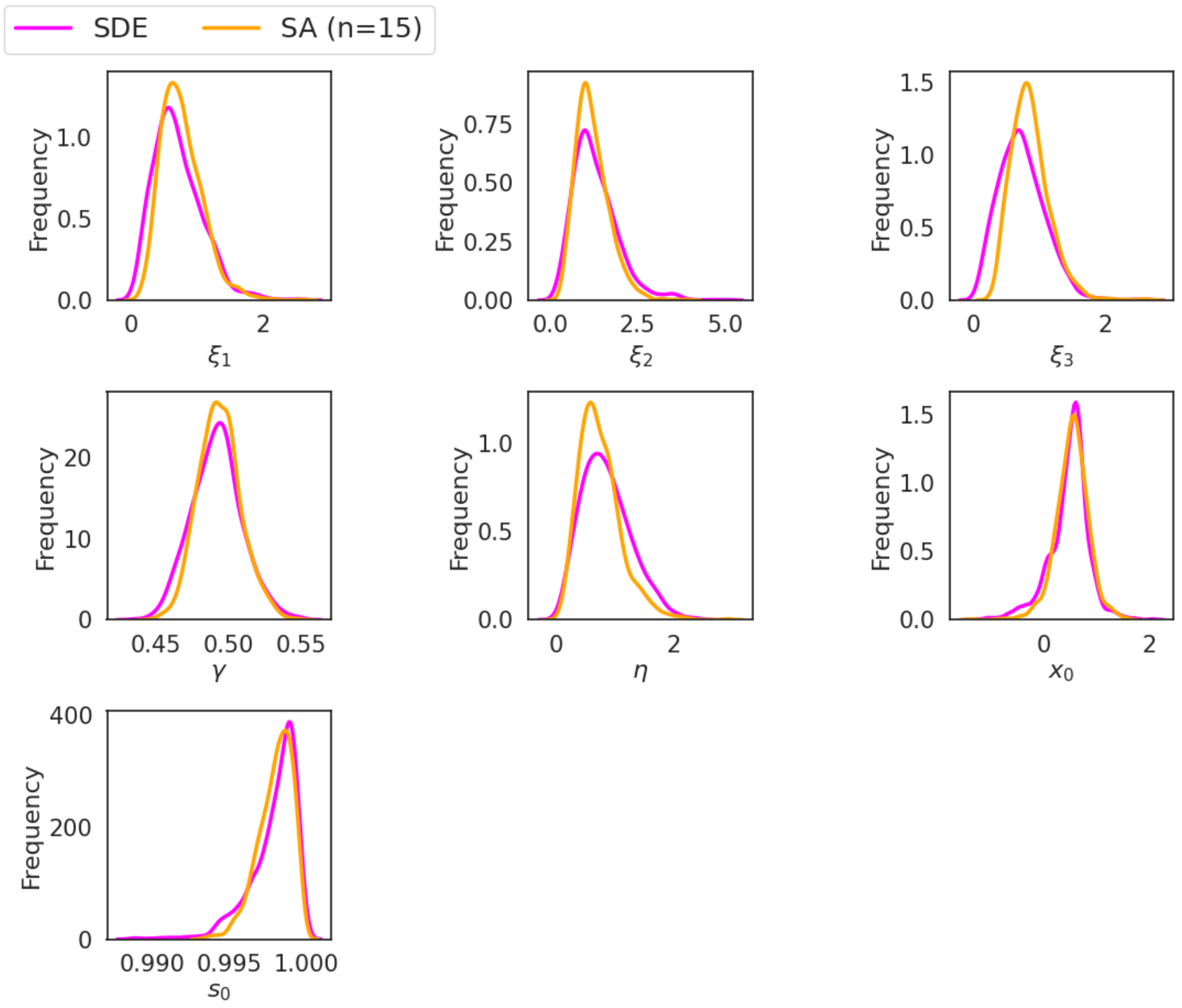

Figure 8: **Considering a negative binomial likelihood for fitting the influenza dataset**: Posterior marginal densities of the parameters.

Appendix J: Further details of the SIRS model parameters

We used the following parameter and initial values, following [10], to generate the simulated dataset: $1/\mu = (50 * 365)$, $1/\alpha = (7 * 365)$, $1/\gamma = 14$, $\beta_0 = 0.65$, $\beta_1 = 0.4$, $\beta_2 = -0.2$, $N = 10000$, $S_0 = 600$, $I_0 = 30$. We placed the following prior for the estimated parameters and initial values: $\beta_0 \sim \mathcal{U}(0.1, 0.7)$, $\sigma \sim \mathcal{U}(0, 0.06)$, $1/\alpha \sim \mathcal{N}(2555, 120^2)$, $1/\gamma \sim \mathcal{N}(14, 1.05^2)$, $S_0 \sim \mathcal{U}(500, 700)$, $I_0 \sim \mathcal{U}(27, 60)$.

In Figure 9 we plot the posterior distribution of the parameters.

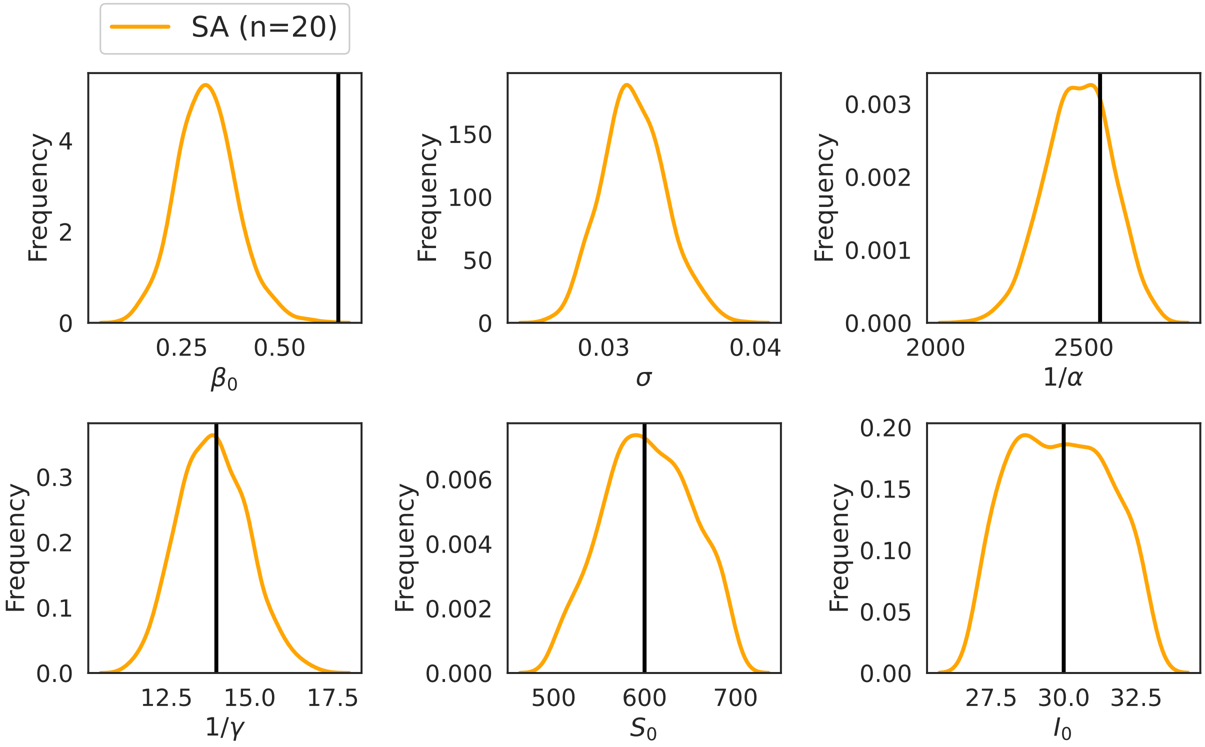

Figure 9: **SIRS model**: Posterior marginal densities of the parameters.

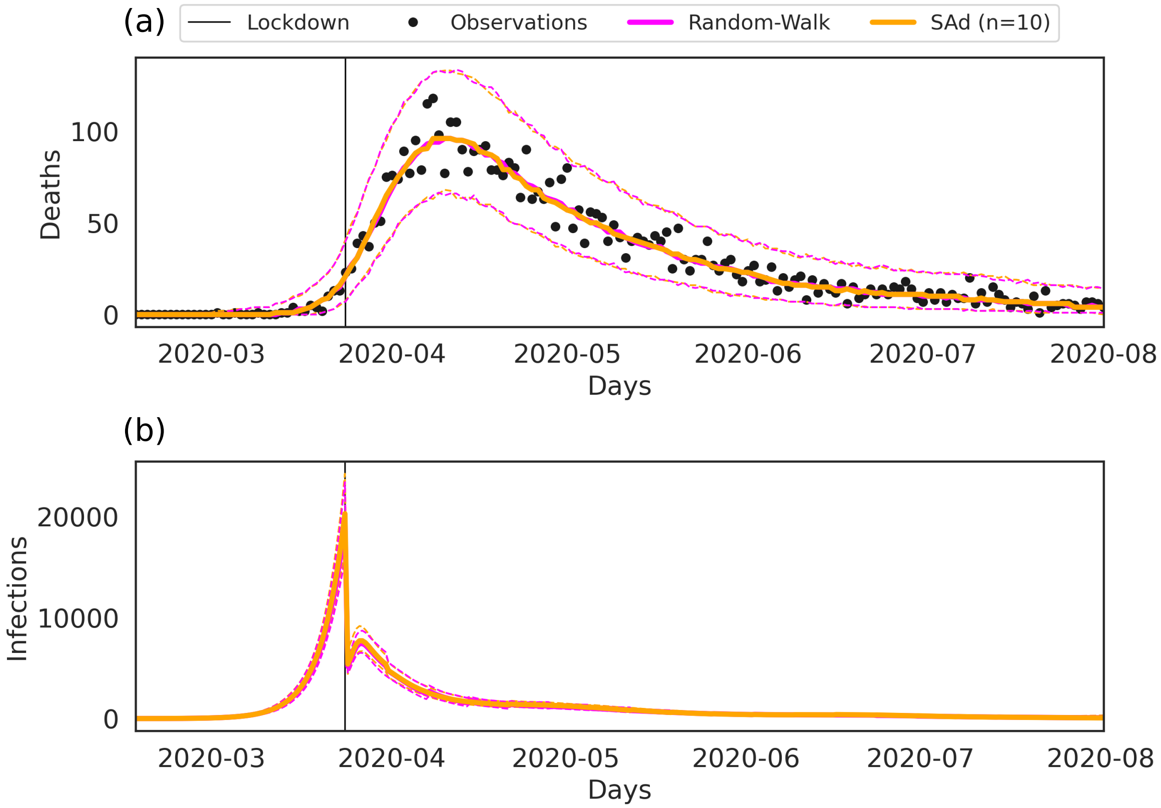

Figure 10: Goodness-of-fit of daily death data (a) and the inferred latent infections (b), produced using the random-walk (magenta lines) and SAd (orange lines) for the region **East of England**. These densities are summarised by the mean (solid lines) and 95% credible intervals (broken lines). The black line indicates the day of lockdown in England 23rd March, 2020.

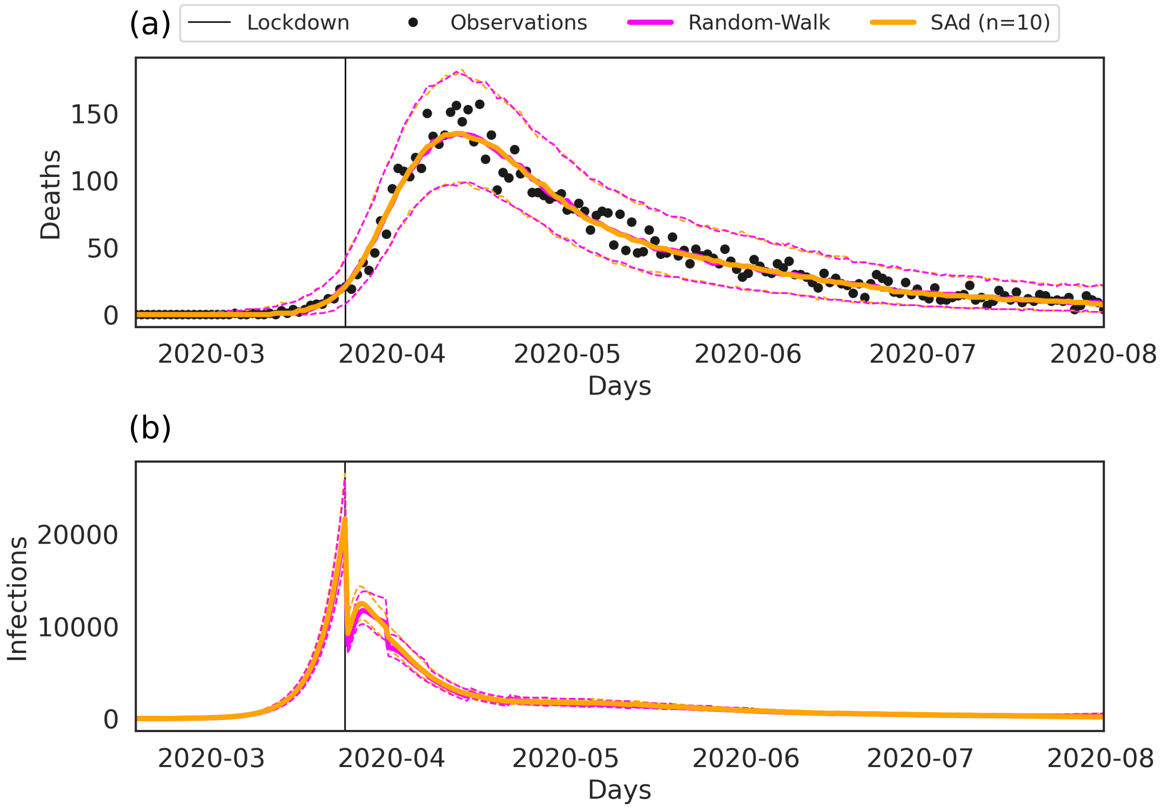

Figure 11: Goodness-of-fit of daily death data (a) and the inferred latent infections (b) for the region **North West**.

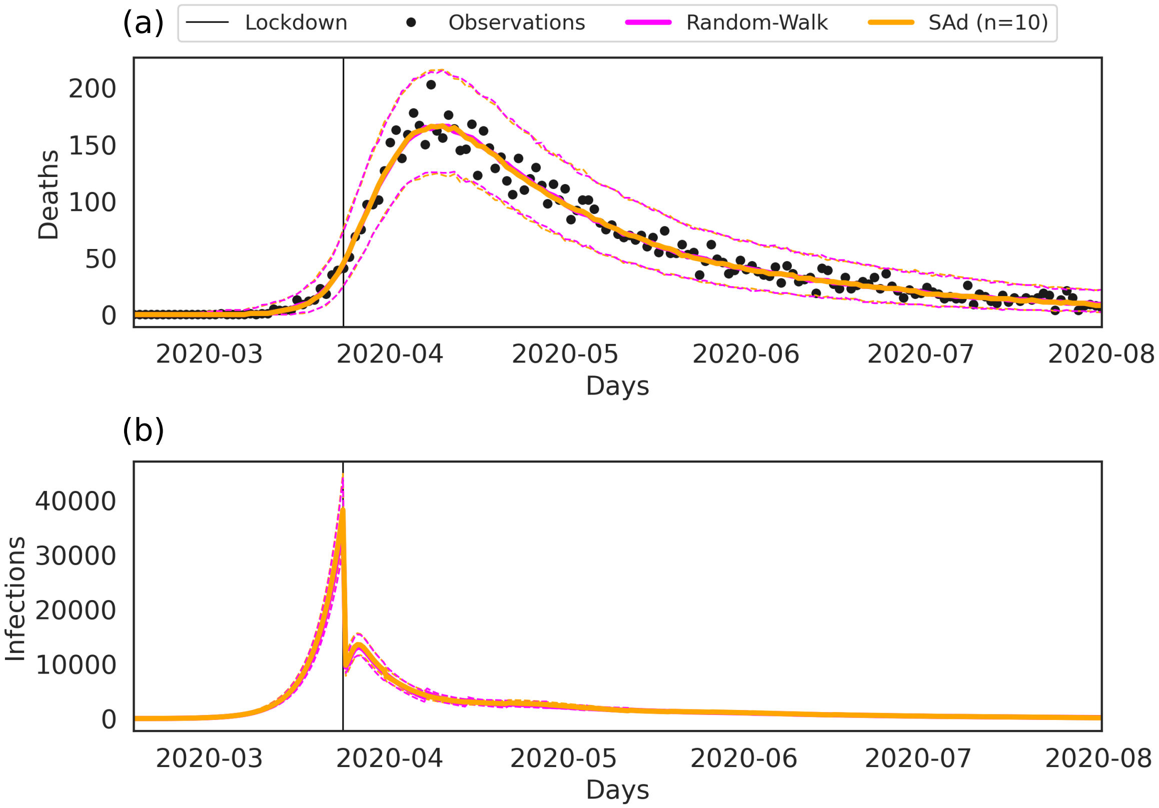

Figure 12: Goodness-of-fit of daily death data (a) and the inferred latent infections (b) for the region **Midlands**.

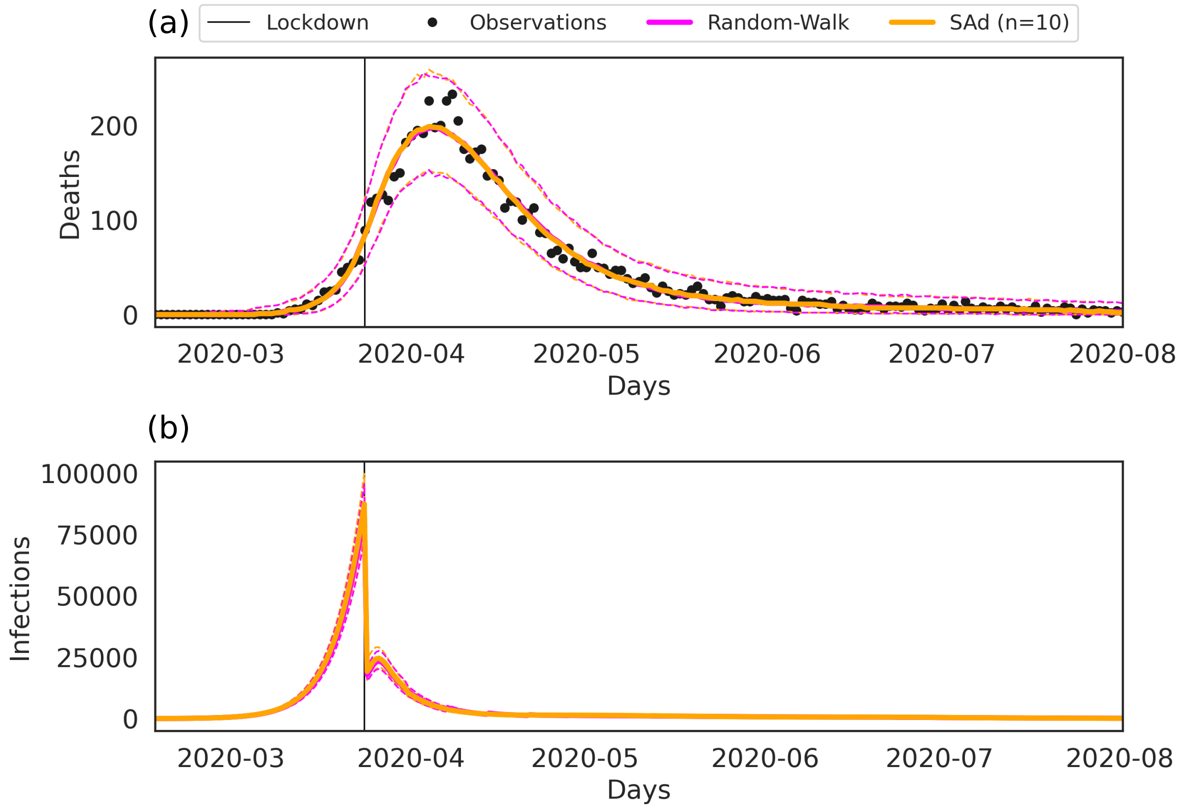

Figure 13: Goodness-of-fit of daily death data (a) and the inferred latent infections (b) for the region **London**.

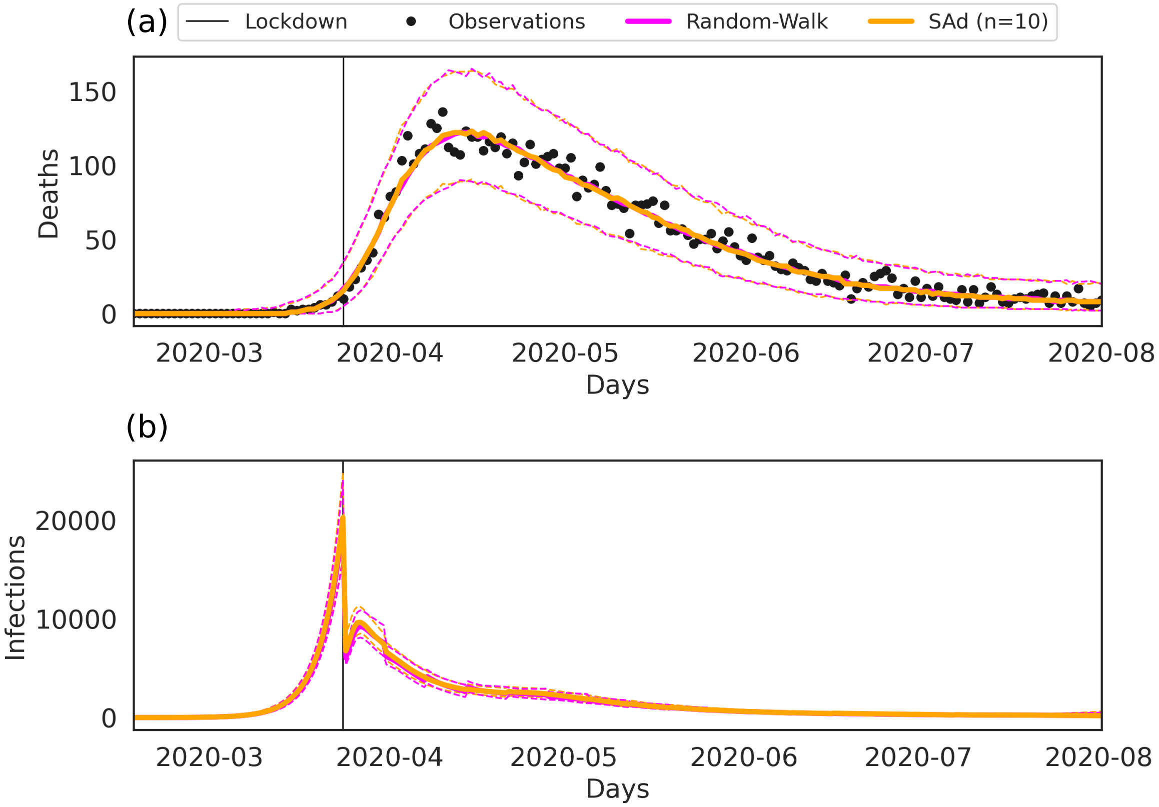

Figure 14: Goodness-of-fit of daily death data (a) and the inferred latent infections (b) for the region **North East and Yorkshire**.

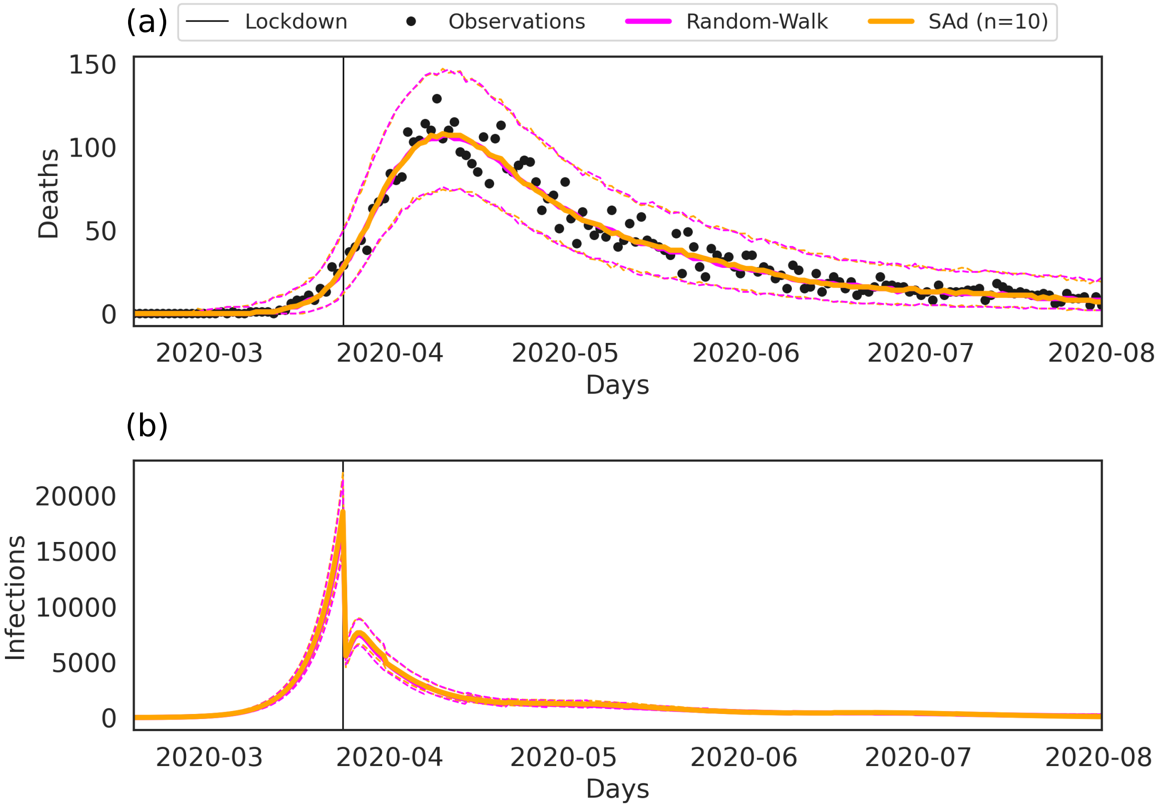

Figure 15: Goodness-of-fit of daily death data (a) and the inferred latent infections (b) for the region **South East**.

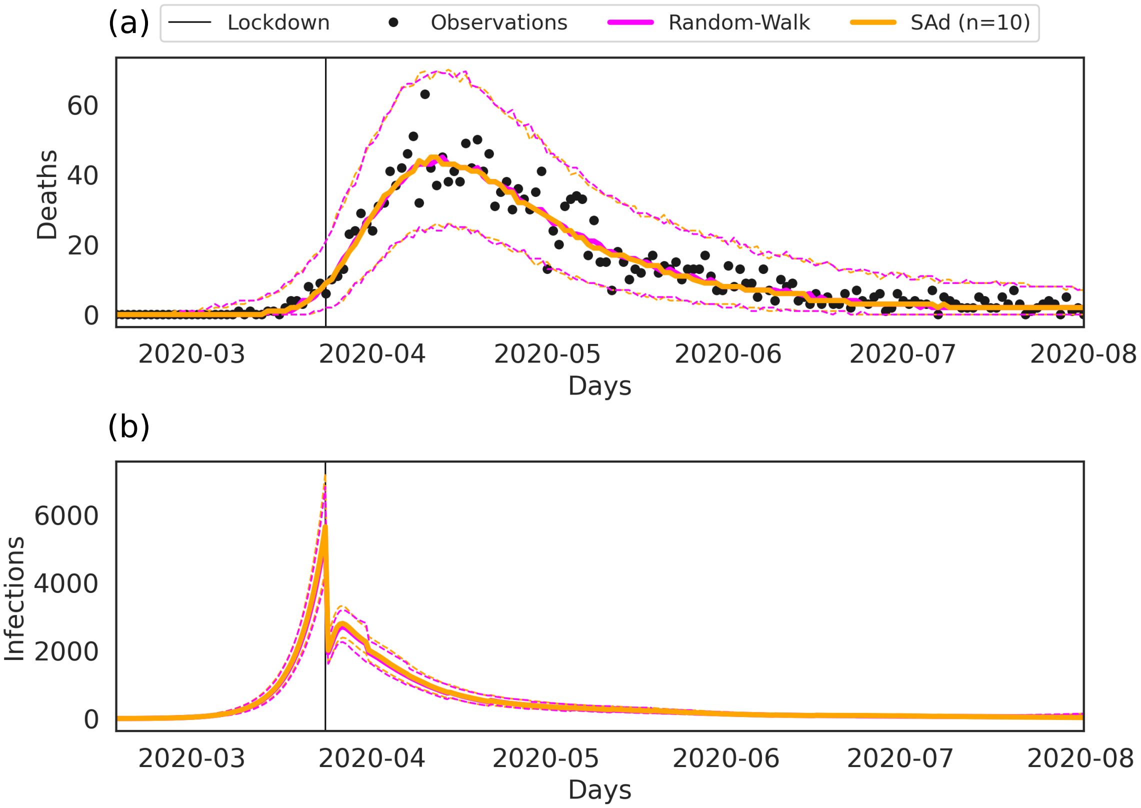

Figure 16: Goodness-of-fit of daily death data (a) and the inferred latent infections (b) for the region **South West**.

References

1. Wuan Luo. *Wiener chaos expansion and numerical solutions of stochastic partial differential equations*. California Institute of Technology, 2006.

2. Simon M. J. Lyons, Amos J. Storkey, and Simo Särkkä. The coloured noise expansion and parameter estimation of diffusion processes. In Peter L. Bartlett, Fernando C. N. Pereira, Christopher J. C. Burges, Léon Bottou, and Kilian Q. Weinberger, editors, *Advances in Neural Information Processing Systems 25: 26th Annual Conference on Neural Information Processing Systems 2012. Proceedings of a meeting held December 3-6, 2012, Lake Tahoe, Nevada, United States*, pages 1961–1969, 2012.

3. Christophe Andrieu and Johannes Thoms. A tutorial on adaptive mcmc. *Statistics and computing*, 18(4):343–373, 2008.

4. Herbert Robbins and Sutton Monro. A stochastic approximation method. *The annals of mathematical statistics*, pages 400–407, 1951.

5. Ross H Johnstone, Eugene TY Chang, Rémi Bardenet, Teun P De Boer, David J Gavaghan, Pras Pathmanathan, Richard H Clayton, and Gary R Mirams. Uncertainty and variability in models of the cardiac action potential: Can we build trustworthy models? *Journal of molecular and cellular cardiology*, 96:49–62, 2016.

6. Helen J Wearing, Pejman Rohani, and Matt J Keeling. Appropriate models for the management of infectious diseases. *PLoS medicine*, 2(7):e174, 2005.

7. Paul Birrell, Joshua Blake, Edwin Van Leeuwen, Nick Gent, and Daniela De Angelis. Real-time nowcasting and forecasting of covid-19 dynamics in england: the first wave. *Philosophical Transactions of the Royal Society B*, 376(1829):20200279, 2021.

8. Krikamol Muandet, Kenji Fukumizu, Bharath Sriperumbudur, and Bernhard Schölkopf. Kernel mean embedding of distributions: A review and beyond. *Foundations and Trends® in Machine Learning*, 10(1-2):1–141, 2017.

9. Arthur Gretton, Karsten M Borgwardt, Malte J Rasch, Bernhard Schölkopf, and Alexander Smola. A kernel two-sample test. *Journal of Machine Learning Research*, 13(Mar):723–773, 2012.

10. Bernard Cazelles, Clara Champagne, and Joseph Dureau. Accounting for non-stationarity in epidemiology by embedding time-varying parameters in stochastic models. *PLoS computational biology*, 14(8):e1006211, 2018.
